# A Comparison of Ci/Gli Activity as Regulated by Sufu in *Drosophila* and Mammalian Hedgehog Response

**DOI:** 10.1371/journal.pone.0135804

**Published:** 2015-08-13

**Authors:** Sekyung Oh, Masaki Kato, Chi Zhang, Yurong Guo, Philip A. Beachy

**Affiliations:** 1 Department of Developmental Biology, Howard Hughes Medical Institute, Stanford University School of Medicine, Stanford, California, United States of America; 2 Department of Molecular Biology and Genetics, Howard Hughes Medical Institute, The Johns Hopkins University School of Medicine, Baltimore, Maryland, United States of America; 3 Department of Biochemistry, Howard Hughes Medical Institute, Stanford University School of Medicine, Stanford, California, United States of America; 4 Division of Pulmonary and Critical Care Medicine, The Johns Hopkins University School of Medicine, Baltimore, Maryland, United States of America; Schulze Center for Novel Therapeutics, Mayo Clinic, UNITED STATES

## Abstract

Suppressor of fused (Su(fu)/Sufu), one of the most conserved components of the Hedgehog (Hh) signaling pathway, binds Ci/Gli transcription factors and impedes activation of target gene expression. In *Drosophila*, the *Su(fu)* mutation has a minimal phenotype, and we show here that Ci transcriptional activity in large part is regulated independently of Su(fu) by other pathway components. Mutant mice lacking *Sufu* in contrast show excessive pathway activity and die as embryos with patterning defects. Here we show that in cultured cells Hh stimulation can augment transcriptional activity of a Gli2 variant lacking Sufu interaction and, surprisingly, that regulation of Hh pathway targets is nearly normal in the neural tube of *Sufu* ^*-/-*^ mutant embryos that also lack *Gli1* function. Some degree of Hh-induced transcriptional activation of Ci/Gli thus can occur independently of Sufu in both flies and mammals. We further note that Sufu loss can also reduce Hh induction of high-threshold neural tube fates, such as floor plate, suggesting a possible positive pathway role for Sufu.

## Introduction

Intercellular signaling mediated by the secreted protein Hedgehog (Hh) critically regulates a multitude of pattern formation and tissue maintenance functions throughout the lifetime of multicellular organisms, and inadequate or inappropriate pathway activity leads to developmental defects or to neoplastic growth [1–4]. Hh ligands activate the pathway by binding to Patched (Ptc) and relieving its suppression of Smoothened (Smo), which in turn causes a family of zinc-finger transcriptional effectors, Cubitus interruptus (Ci) in *Drosophila* and the Gli proteins in vertebrates, to switch from transcriptional repressors to activators of Hh-target genes [1,2,4]. As a result, transcriptional activity of Ci/Gli is precisely regulated in proportion to Hh stimulus to elicit the appropriate cellular response [5].

Whereas in *Drosophila* all Hh-dependent transcriptional activation and repression functions are carried out by Ci, in mammals these functions are subdivided among three Gli proteins [5]. Thus, although Gli2 and Gli3 each can be found in full-length or proteolytically processed forms [6–9], transcriptional activation is primarily executed by full-length Gli2, and transcriptional repression by the processed form of Gli3 [9–11]. Gli1 is nonessential [12] but is a target of pathway activity and a positive transcriptional effector, and thus functions as an amplifier of the activated state [10,13]. Pathway activation ultimately results in Gli-mediated induction of transcriptional targets, including *Ptc* and *Gli1* [14].

Many core elements of the Hh signaling pathway were identified through genetic approaches in *Drosophila*, and in general the mammalian orthologs of these proteins are required for similar aspects of vertebrate Hh signal transduction [1,2]. For example, in flies, the kinesin-related protein Costal2 (Cos2) interacts with Ci to inhibit its nuclear translocation [15,16], and also promotes proteolytic processing of Ci to generate a transcriptional repressor form (CiR) [17]. Cos2 thus acts to suppress Ci-mediated transcriptional activity in the absence of Hh stimulation, and *cos2* mutants show some degree of constitutive pathway activation. Cos2 also contributes, however, to full activation of Ci-mediated transcription [16,18,19], acting with its tightly bound partner, Fused (Fu), an apparent serine/threonine kinase, which is essential for Hh signaling [20]. Like *cos2*, the mammalian ortholog *Kif7* also appears to act positively and negatively in Gli protein modulation. Thus, for example, a negative regulatory role for *Kif7* is revealed by the dorsal expansion of ventral cell fates within the developing neural tube upon loss of *Kif7* function [21,22]. However, a positive role for *Kif7* is also suggested in certain genetic backgrounds by exacerbation of pathway loss-of-function phenotypes or partial rescue of pathway gain-of-function phenotypes when *Kif7* function is additionally removed [21]. Thus, at the level of their overall impact on Hh pathway activity, Cos2 and Kif7 in flies and mammals appear to function similarly.

Despite the conservation of sequence and apparent function of many components of the Hh signaling pathway between invertebrates and vertebrates, the functional roles of other pathway components appear to have diverged considerably [1,2]. In *Drosophila*, the Fused (Fu) kinase has an essential role for pathway activation [20]. The *fu* mammalian ortholog, however, is not essential for Hh signaling and instead is important for the formation of motile cilia [23–25]. Along the same lines, Suppressor of fused (Sufu), which binds directly to Ci/Gli and impedes transcriptional activation of target genes [26–31], appears to play a central role in the mammalian pathway [32,33] but is nearly dispensable for pathway function in *Drosophila* [34]. Indeed, *Su(fu)* mutant flies show a nearly undetectable phenotype, but loss of *Su(fu)* almost perfectly suppresses the lethality and patterning phenotypes of mutations of the *fu* gene [34]. Thus the negative role of Su(fu) in *Drosophila* pathway regulation is revealed primarily by the suppression of *fu* mutant phenotypes upon loss of *Su(fu)* function [34]. On the other hand, mutant mice lacking Sufu function die as embryos with cephalic and neural tube defects that mirror the phenotype resulting from deletion of the mammalian Hh receptor Ptch1 [32,33]. Thus, loss of murine Sufu function results in severe ectopic activation of Hh signaling, suggesting that Sufu is a critical negative regulator of Hh signaling in the mouse.

The striking differences in the phenotypes of fly *Su(fu)* and mammalian *Sufu* mutants suggest that the function of Sufu homologs within the Hh pathway has diverged dramatically in vertebrate and invertebrate evolution [2]. Such a divergence is particularly surprising given that Sufu is one of the most conserved components in Hh pathway response, with 38% identity compared to that of Ptch/Ptc (23%), Smo (33.7%) and Gli2/Ci (24.2%). This conservation of Sufu extends to well-established direct physical interactions with all three Gli proteins as well as Ci [26–31], and physical dissociation of mammalian Gli proteins from Sufu has been proposed as a central feature of pathway activation [35,36]. Consistent with such a mechanism, recent structural studies revealed a conformational change of human SUFU protein, likely induced by Hh stimulation, that leads to decreased interaction between Sufu and a well-conserved SYGHL motif conserved in Ci/Gli proteins [37,38].

In this study, we investigate the role of Sufu in Ci/Gli modulation in flies and mammals, and ask how important Sufu suppression of basal Ci/Gli activity is for achieving pathway regulation in each of these organisms. In *Drosophila*, we provide evidence that phosphorylation of Su(fu) is irrelevant for pathway regulation, and that Fu kinase together with other pathway components increase Ci transcriptional activity independently of Su(fu). On the other hand, *Sufu* homozygous mutations in mice are embryonic lethal, with a phenotype resembling that of *Ptch* homozygous mutants. However, the interpretation of this *Sufu* phenotype is complicated because Hh pathway output is a result of the combined activities of Gli activators and repressors, the protein levels of which are altered by loss of *Sufu* function [36,39]. To simplify this complexity we genetically removed *Gli1* function from *Sufu* mutant mice, and found that loss of *Gli1* function partially rescues the *Sufu* mutant phenotype, as seen from the dramatic restoration of neural tube patterning of double mutants as compared to *Sufu* single mutants. These results thus indicate that some degree of Ci/Gli regulation can occur in the absence of Sufu suppression in flies and mammals.

## Materials and Methods

### *Drosophila* lines

*Drosophila melanogaster* lines used include: *ptc-lacZ* [40], *C765-Gal4* [41], and *UAS-Su(fu)* [42] from K. Basler, *UAS-Ci* and *UAS-CiZnC* from R. Holmgren [43]. *fu* ^*mH63*^ [44] and balancer lines from Bloomington *Drosophila* Stock Center (Bloomington, IN). *ptc-lacZ/CyO-krGFP; C765-Gal4* males were crossed to virgin females of either: 1) *UAS-Ci*, *UAS-Su(fu); UAS-Ci*, *2) fu* ^*mH63*^ */FM7c-krGFP*; *UAS-Ci*, 3) *UAS-CiZnC*, 4) *UAS-Su(fu); UAS-CiZnC*, or 5) *fu* ^*mH63*^ */FM7c-krGFP*; *UAS-CiZnC*. Wing imaginal discs were dissected from third instar larvae that did not express GFP, and processed for immunostaining.

### Mouse strains

The animal procedures carried out for this work were approved by the Administrative Panel on Laboratory Animal Care at Stanford University. *Sufu* [32], and *Gli1-LacZ* [10] mutant mice were from Jackson Laboratory and were genotyped as following the manufacture’s instruction. The primer sets for *Sufu* mutant embryos were used as described [32]. X-gal staining and immunocytochemistry were performed as described [10,45].

### Antibodies

Anti-Su(fu) (25H3) and anti-Fu (26F11) mouse monoclonal antibodies (mAbs) and anti-Cos2 rabbit polyclonal antiserum have been previously described [46]. Other antibodies used are: anti-Ci (2A1) rat mAb (a gift from R. Holmgren), rabbit anti-Lamin Dm0 (R836) (a gift from P. Fisher), anti-β-Tubulin mAb (E7) (Developmental Studies Hybridoma Bank), rat anti-HA (3F10) (Roche), mouse anti-HA (HA11) (Covance), mouse anti-V5 (Invitrogen), mouse anti-Myc (9E10), rabbit anti-Myc (A14) and rabbit anti-Sufu (Santa Cruz Biotechnology), mouse anti-FLAG (M2), mouse anti-acetylated α-Tubulin (Sigma), mouse anti-β-galactosidase (Promega), rabbit anti-GFP (Invitrogen), mouse anti-Nkx2.2, Foxa2 and Isl1/2 (Developmental Studies Hybridoma Bank), rabbit anti-Pax6 (Covance), guinea pig anti-Gli2 (a gift from J. Eggenschwiler), and rabbit anti-Gli2 (a gift from B. Wang).

### Plasmids and RNA inteference

Expression constructs used in *Drosophila* cultured cells were constructed in the pAcSV vector [47]. Various mammalian expression constructs of mGli2, pCEFL3xHA-Gli2s were constructed using PCR as described previously [48]. The retroviral hSUFU and 3xHA-tagged Gli2 (WT and ΔSufu) constructs were constructed in the pMSCV (Clontech) and MigR1 vector [49], respectively. RNAi targeting of *Drosophila* Hh pathway components and shRNA-mediated knockdown of mouse Dync2h1 were as previously described [15,48].

### Luciferase reporter assay

*ptc*-luciferase assays in cl-8 cells and 8XGli-binding site luciferase assays in NIH3T3 cells were as previously described [15,48]. For reconstitution assays in S2R+ cells, 1:1 (w/w) ratio of expression constructs were transfected either in 6-well plate format for Western analyses or in 24-well plate format together with *ptc* luciferase and *copia Renilla* luciferase constructs for *ptc*-luciferase assays.

### Cell culture, co-immunoprecipitation assays, and phosphorylated peptide analysis

Cl-8 cells were cultured as previously described [15]. S2R+ cells were cultured in S2 medium supplemented with 10% FBS and penicillin/streptomycin. HEK293F cells (Invitrogen) and *Gli2*,*3* double knockout (*Gli2* ^*-/-*^ *; Gli3* ^*-/-*^) mouse embryonic fibroblasts (MEFs) [50] were cultured in DMEM supplemented with 10% fetal bovine serum (FBS), penicillin/streptomycin, and L-glutamine. NIH3T3 cells were maintained in DMEM supplemented with 10% calf serum, penicillin/streptomycin, and L-glutamine. *Sufu* ^-/-^ MEF cells [33] were cultured in DMEM supplemented with 10% fetal bovine serum, 10 μg/ml gentamicin, penicillin/streptomycin, and L-glutamine. For co-immunoprecipitation (co-IP) experiments, cells were lysed in PBS supplemented with 1.0% Triton X-100, phosphatase inhibitors (1 mM Na_3_VO_4_ and 50 mM NaF), and Complete Mini Protease Inhibitor (Roche). For the other gel-based experiments without IP reactions, cells were lysed in RIPA buffer (50 mM Tris-HCl (pH 7.5), 150 mM NaCl, 1% Triton X-100, 0.5% Na-deoxycholate, and 0.1% SDS) supplemented with Complete Mini Protease Inhibitor (Roche). Antibody matrices covalently attached to either sepharose or agarose were used for all co-IP reactions. Anti-Su(fu) (25H3) mAb affinity matrix was generated as previously described [46]. Purification and analysis of phosphorylated peptides following the large scale immunoprecipitation were performed as described [51].

### Retroviral infection and flow cytometry

Stable *Sufu* ^*-/-*^ MEFs with integrated vector expressing human SUFU (hSUFU) were generated by retroviral infection and selection with 3.0 μg/mL of puromycin. The viral supernatant was harvested from MSCV-hSUFU-transfected Phoenix ampho packaging cells. *Sufu* ^*-/-*^ MEFs were plated at a density of 8x10^5^ cells/well of 10 cm dish and 24 h later, cells were infected with the viral supernatants containing 6 μg/mL of polybrene (Sigma). *Gli2* ^*-/-*^ *; Gli3* ^*-/-*^ MEFs expressing HA-Gli2 or Gli2ΔSufu were generated by retroviral infection and selection of GFP positive cells using Flow Cytometry (BD digital Vantage). The viral supernatant was harvested from MIG-HA-Gli2 or Gli2ΔSufu (IRES-GFP)-transfected Phoenix ampho packaging cells.

### Quantitative real time PCR

Total RNA from *Gli2* ^*-/-*^ *; Gli3* ^*-/-*^ MEFs with or without HA-Gli2 or Gli2ΔSufu stable integration was extracted using Trizol (Invitrogen) as recommended by the manufacturer. Quantitative real time PCR (qRT-PCR) was performed using the QuantiTect SYBR Green PCR Kits (Qiagen). Reactions were carried out in an Applied Biosystems 7300 Real Time PCR system (Applied Biosystems). Primer sets for *Gli1* and *Hprt1* gene were described previously [52]. The *Hprt1* gene was used as an internal control. The threshold cycle (CT) for *Gli1* was first normalized to the corresponding *Hprt1* CT. Relative fold differences were then determined using the 2^–ΔΔCT^ method [53].

### Immunofluorescence microscopy

Cells were fixed in 4% formaldehyde for 20 min. Cells were permeabilized with PBS/0.2% Triton X-100 for 5 min and nonspecific binding sites were blocked with 1.5% normal goat serum (NGS) in PBST (PBS with 0.1% Tween-20). Cells were stained with primary antibodies diluted in 1.5% NGS/PBST for 1 h at room temperature. Appropriate Alexa 488-, Alexa 594-conjugated secondary antibodies were used.

## Results

### Fu and Cos2 are required to overcome Su(fu) suppression in *Drosophila*

**Fig 1:**
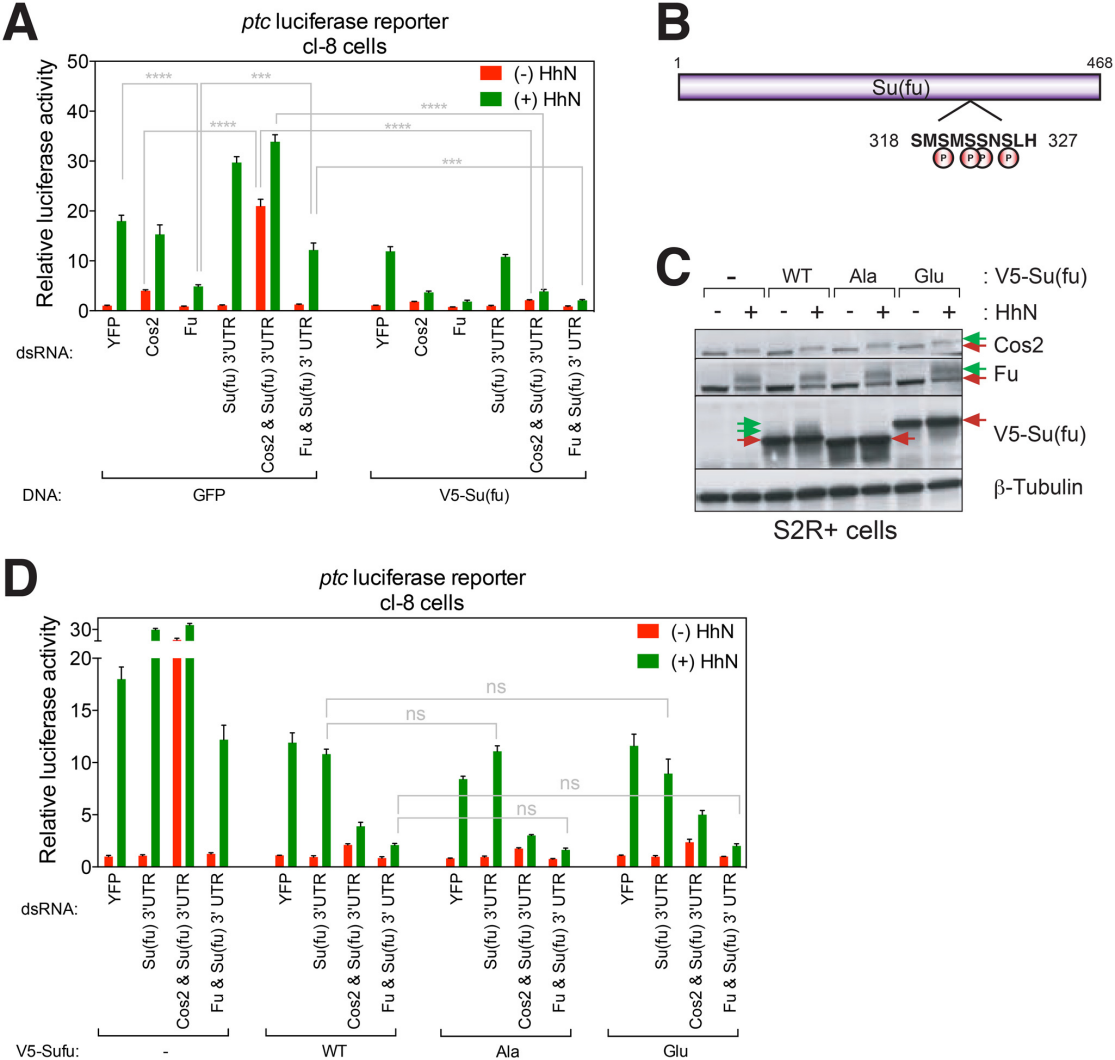
Functional characterization of Su(fu) phosphorylation in Drosophila. (A) Hh-dependent transcriptional activation was measured by activity of a *ptc*-luciferase reporter normalized to constitutively expressed control *Renilla* luciferase. Cells were stimulated with or without conditioned medium containing HhN, the N-terminal signaling domain of the Hh protein. (B) Identification of phosphorylation sites in Su(fu). A schematic diagram of Su(fu) is shown and phosphoserine residues identified by mass spectrometry in endogenous Su(fu) purified from HhN-stimulated *Drosophila* cl-8 cells are marked with “P”. (C) Western blot analysis of Ala or Glu substitution of Su(fu) phosphoresidues. (D) Endogenous *Su(fu)* mRNA was targeted using dsRNA corresponding to the 3’ untranslated region (3’UTR), and expression constructs encoding Su(fu) but lacking the 3’UTR sequence were used to test for function of Su(fu) variants, with pathway activity measured as in (A). A representative experiment from at least three independent experiments is shown. Error bars show mean +/- standard deviation. Statistical significance was measured by Student’s *t*-test: **** (P<0.0001), *** (0.0001<P<0.001), and ns (not significant, P>0.05).

In *Drosophila*, although *Su(fu)* mutations alone show only a subtle phenotype, they completely suppress the lethality and strong patterning defects of mutations in the *fu* gene, which is essential for normal Hh signaling [20,34]. Thus, *fu* function is not required for Hh pathway activation when Sufu is absent suggesting that Fu plays a role in relieving suppression by Su(fu). To facilitate further analysis of *fu* and *Su(fu)*, we tested the function of these components in pathway activity in cl-8 cultured cells using a *ptc*-luciferase reporter to monitor Hh-stimulated transcriptional activity [15,46]. Consistent with previous mutational analysis in *Drosophila* [20,34], RNAi against *fu* caused a loss of pathway activity whereas RNAi targeting of *Su(fu)* via its 3’UTR produced a mild increase in unstimulated as well as stimulated pathway activaty (Fig 1A). Also consistent with previously reported suppression of *fu* mutations by *Su(fu)* mutations [34], combined RNAi targeting of *fu* and *Su(fu)* rescued pathway activity to a level similar to that of the control YFP RNAi (Fig 1A). Restoration of wild-type (WT) *Su(fu)* function via expression of an exogenous Su(fu) construct reversed this rescue and produced loss of pathway activity like that produced by RNAi against *fu* alone (Fig 1A).

Also consistent with *in vivo* observations [16], we further observed that RNAi against endogenous *Su(fu)* dramatically enhanced the partial pathway-activating effect of *cos2* RNAi, particularly the constitutive reporter expression in the absence of Hh stimulation (Fig 1A). Under these circumstances, restoration of WT Su(fu) expression dramatically suppressed both the constitutive and Hh-induced reporter expression (Fig 1A). Loss of either Cos2 or Fu thus appears to render cells extraordinarily sensitive to the presence or absence of Su(fu), and these *in vitro* assays thus reproduce and provide quantitative assays of Su(fu) behavior *in vivo*. These results are consistent with the previously reported strong physical interaction of Fu and Cos2 [19,54,55] and suggest that the Fu-Cos2 complex is required to overcome Su(fu) suppression.

### No functional effect of Su(fu) phosphorylation in *Drosophila*

In *Drosophila*, Hh stimulation induces phosphorylation of Su(fu), which decreases with loss of Fu [19,56]. In addition, Fu can physically interact with Su(fu) [54]. Thus, the prevailing view has been that Fu kinase increases Ci transcriptional capability by phosphorylating and inactivating Su(fu) [1,57]; nevertheless, the dispensability of Su(fu) for normal *Drosophila* development [34] also suggests that inactivation of Su(fu) cannot be the sole mechanism of Ci activation.

To evaluate the model of Su(fu) inactivation by Fu-mediated phosphorylation, we identified phosphorylated amino acid residues on Su(fu) and tested their functional significance via mutational analysis. Using a matrix with immobilized monoclonal antibody against Su(fu) [19], we purified Su(fu) protein from lysates of cl-8 cells. Mass spectrometry analysis in tandem with immobilized metal affinity chromatography (IMAC) separation identified four phosphorylated serine residues in Su(fu), S320, S322, S323, and S325, which are poorly conserved among Su(fu) homologues of other species (Fig 1B and S1 Fig). We altered all four serine residues either to alanine (Su(fu)-Ala) or to glutamate (Su(fu)-Glu) to block or mimic phosphorylation, respectively. The V5-tagged Su(fu)-Ala (V5-Su(fu)-Ala) and Su(fu)-Glu (V5-Su(fu)-Glu) proteins expressed in *Drosophila* embryo-derived S2R+ cells migrated faster and slower, respectively, in Western blot analysis following SDS-PAGE as compared to WT V5-Su(fu) (Fig 1C). In addition, although WT Su(fu) in Hh-stimulated cells shows heterogeneity in its electrophoretic migration due to phosphorylation [19] (Fig 1C), both V5-Su(fu)-Ala and V5-Su(fu)-Glu were resolved as single bands in either the presence or absence of HhN stimulation (Fig 1C), suggesting that these alterations block Hh-induced changes in Su(fu) phosphorylation and that we have identified most, and possibly all, Su(fu) phosphoresidues.

In all these RNAi conditions, expression of V5-Su(fu)-Ala or V5-Su(fu)-Glu exerted nearly identical effects to those of WT V5-Su(fu) on the activity of the *ptc*-luciferase reporter (Fig 1D). Thus, although other as yet unidentified phosphorylated residues might be critical for Su(fu) function, the majority of Su(fu) phosphorylation resolvable in SDS-PAGE does not appear to cause a significant gain or loss in the pathway-suppressing activity of Su(fu) detected in our cultured cell assays, which closely reproduce the behavior of Su(fu) in *Drosophila*.

### Distinct regions in Fu bind to Su(fu) and Cos2

**Fig 2:**
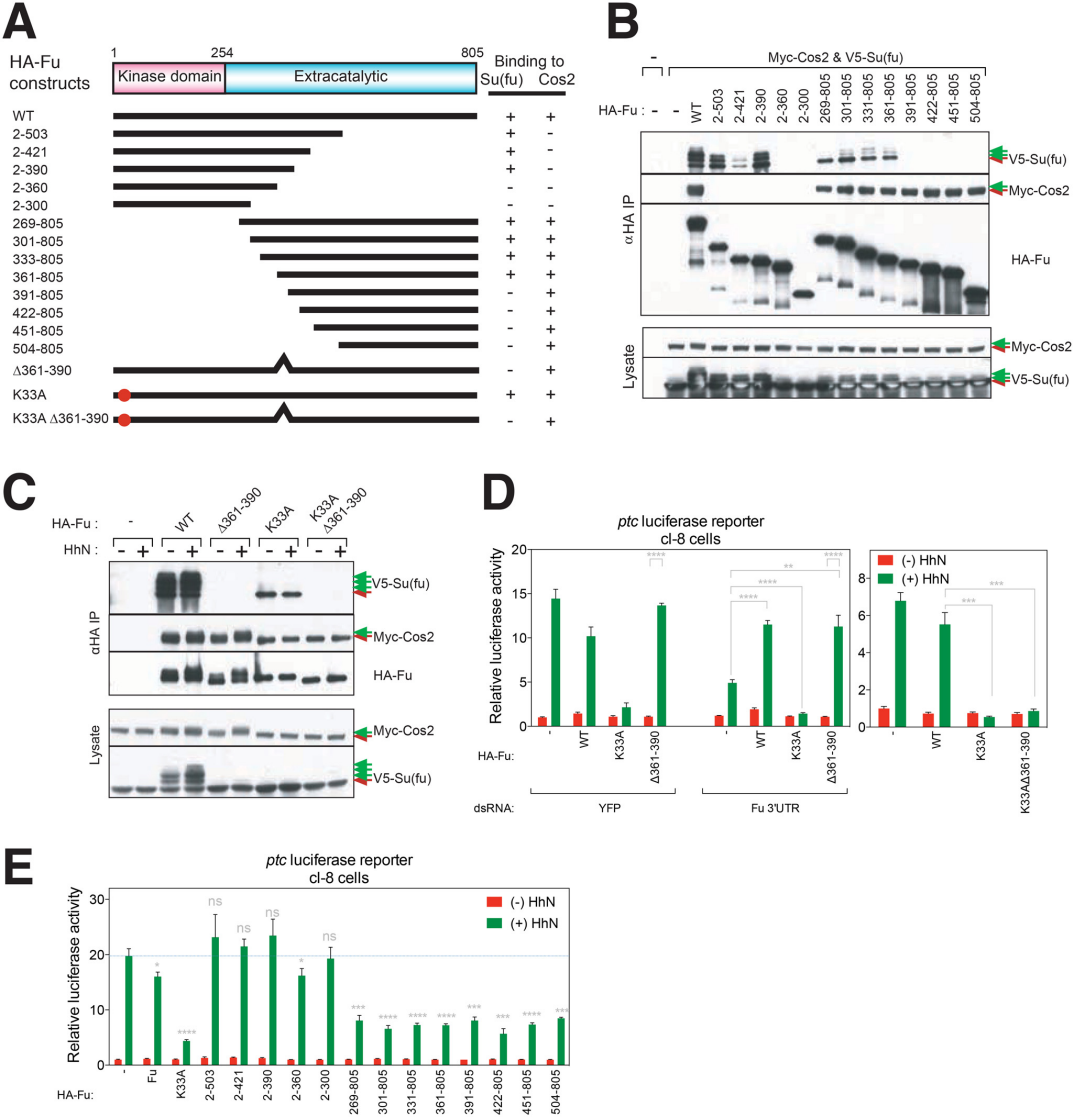
Physical and functional characterization of Fu interaction with Sufu and Cos2. (A) Schematic representation of Fu variants and their interactions with Su(fu) and Cos2. (B) Distinct regions in Fu bind to Su(fu) and Cos2. S2R+ cells were transiently co-transfected with HA-tagged Fu variants (A) and Myc-tagged Cos2 and V5-tagged Su(fu) constructs. Green arrows denote phosphorylated forms of Su(fu). (C) Su(fu) phosphorylation (green arrows) does not increase in response to Hh in the presence of either kinase-inactive Fu or Fu lacking Su(fu) binding determinants (Δ361–390). (D) Fu kinase functions in Hh-dependent transcriptional activation in cl-8 cells independently of Su(fu) binding determinants. Expression constructs encoding Fu but lacking the 3’UTR sequence were used to test for rescue of Fu function. (E) Fu variants containing Cos2-interacting determinants but lacking kinase activity suppress Hh-dependent transcriptional activity. A representative experiment from at least three independent experiments is shown. Error bars show mean +/- standard deviation. Statistical significance was measured by Student’s *t*-test: **** (P<0.0001), *** (0.0001<P<0.001), ** (0.001<P<0.01), * (0.01<P<0.05), and ns (not significant, P>0.05). P values in (E) derive from the HhN-induced mediated by each Fu variant as compared to luciferase activity with no Fu expression (blue dotted line).

We used a series of Fu deletions to map the interactions between Fu and Su(fu), and between Fu and Cos2 using a co-immunoprecipitation (co-IP) assay. We constructed N-terminally HA-tagged full-length Fu (HA-Fu) and a series of nested truncations from either the N- or the C-terminus (Fig 2A), and co-transfected them into S2R+ cells with V5-Su(fu) and Myc-tagged Cos2 (Myc-Cos2). As a Fu fragment can form a hetero-dimer with full-length Fu [58], V5-Su(fu) and Myc-Cos2 might be able to interact with the nested HA-Fu truncations indirectly through binding to endogenous Fu. To minimize this effect, Fu 3’UTR dsRNA was also co-transfected to knock down endogenous Fu. We observed that IP using an anti-HA matrix efficiently retrieved Myc-Cos2 when either WT or any truncated HA-Fu construct that contains amino acid residues 504–805 (aa504-805) was expressed (Fig 2A and 2B). HA-Fu also interacted weakly with V5-Su(fu) (Fig 2A and 2B) and this weak interaction persisted with any HA-Fu truncation that retained aa361-390 (Fig 2A and 2B).

We additionally constructed a small deletion variant of HA-Fu that lacks aa361-390 (FuΔ361–390), and tested its binding to either Myc-Cos2 or V5-Su(fu) as above. Whereas Myc-Cos2 binding was preserved with FuΔ361–390, V5-Su(fu) binding was lost (Fig 2A and 2C). We also deleted aa361-390 in the context of Fu^K33A^, a *trans*-dominant kinase-inactive form of Fu [59], and found that the resulting Fu^K33A^Δ361–390 showed normal binding to Myc-Cos2 but not to V5-Su(fu) whereas Fu^K33A^ bound to both Myc-Cos2 and V5-Su(fu) with or without Hh stimulation (Fig 2A and 2C). We conclude that Fu binds to Su(fu) and to Cos2 through distinct regions in the extracatalytic domain, (aa361-390 and aa504-805, respectively), and that disruption of Fu kinase activity does not affect the ability of Fu to bind to Su(fu) and Cos2.

### Fu kinase mediates transcriptional activation independently of Su(fu) binding

We tested the significance of Fu-Su(fu) interaction using WT Fu and the small deletion variant FuΔ361–390. When expressed with the control YFP RNAi, they both showed normal response to Hh stimulation (Fig 2D). As also shown above, RNAi against either *fu* 3’UTR or its coding region lowered *ptc*-luciferase activity; this loss of response was fully rescued by expression of either WT Fu or FuΔ361–390 (Fig 2D). In contrast, Fu^K33A^ failed to rescue and actually further suppressed *ptc*-luciferase activity (Fig 2D), indicating that Hh pathway activation by Fu requires kinase activity. The kinase-inactive Fu variant lacking interaction with Su(fu) (Fu^K33A^Δ361–390) was identical in its ability to suppress the pathway activity (Fig 2D). The interaction of Fu with Su(fu), mediated by Fu aa361-390 thus appears to be inconsequential with respect to Fu activity.

A previous study showed that expression in the *Drosophila* wing of a fragment of the Fu extracatalytic domain consisting of amino acid residues 422–805 phenocopied the loss of Fu by interfering with binding of endogenous Fu to Cos2 [60]. Similarly, we observed that expression of an HA-tagged form of this same fragment (Fu422-805) in cl-8 cells significantly reduced Hh-induced *ptc*-luciferase activity (Fig 2E). We were able to further narrow the region in Fu that produces the pathway-suppressing effect to amino acid residues 504–805 (Fig 2E), which corresponds to the Cos2-binding region as defined above (Fig 2A and 2B). In contrast, expression of HA-Fu constructs that contain the Su(fu)-binding region but not the Cos2-binding region (Fu2-390) did not result in reduction of *ptc*-luciferase activity (Fig 2E).

These results in aggregate show that regulation of pathway activity by Fu is not dependent on interaction with Su(fu) but instead requires a region of Fu known to interact with Cos2 [61,62]. We noted that phosphorylation of Su(fu) is dramatically reduced by deletion from Fu of the Su(fu)-interacting region (Fig 2C; FuΔ361–390) without affecting Hh pathway activity, consistent with our previous conclusion that phosphorylation of Su(fu) may not critically affect its function. Curiously, we found that Su(fu) phosphorylation was enhanced by some forms of Fu that retain the Su(fu)-interacting region but lack the kinase domain (Fu301-805, Fu331-805, Fu361-805; Fig 2B), suggesting the possibility that Fu may enhance Su(fu) phosphorylation by mediating interactions with other kinase(s). These results are consistent with and support the conclusion that Fu mediates transcriptional activation independently of an interaction with or effect on Su(fu).

### A cellular reconstitution system for analysis of Ci structure and function

**Fig 3:**
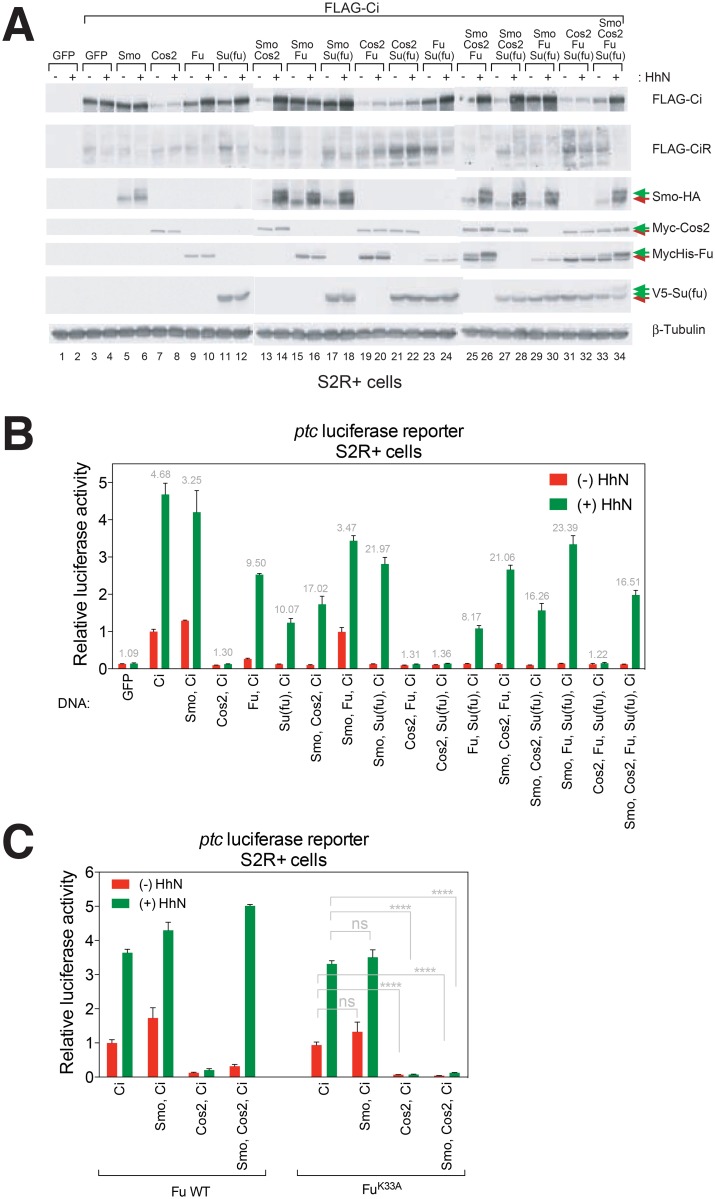
Reconstitution of Hh signaling in S2R+ Cells. (A) Western blot analysis of exogenously introduced pathway components. Permuted combinations of pathway components reveal their effects on posttranslational modifications of themselves or other components. Hh-induced phosphorylation of pathway components are indicated by green arrows. β-Tubulin is used as a loading control. (B) Hh pathway activity was measured by expression of luciferase under control of the Hh-responsive *ptc* promoter (*ptc*-luciferase) and of the control *Renilla* luciferase under the ubiquitous *copia* promoter (*copia*-*Renilla* luciferase). Gray numbers indicate fold induction of luciferase activity in response to HhN stimulation. (C) Fu kinase-dependent Ci activation in response to HhN occurs in the context of a Hh-responsive complex comprising Smo, Cos2, Fu and Ci. A representative experiment from at least three independent experiments is shown. Error bars equal to mean +/- standard deviation. Statistical significance was measured by Student’s *t*-test: **** (P<0.0001) and ns (not significant, P>0.05).

Although Fu-Su(fu) physical interaction appears not to be important for pathway activation (Fig 2D), Fu could affect how Su(fu) regulates Ci, thereby altering pathway activity indirectly. To address the question of whether pathway activation by Fu depends on Su(fu) suppression of Ci, we developed a cellular reconstitution system in S2R+ cells, which lack endogenous Ci expression but retain the posttranslational changes of most other upstream pathway components in response to Hh [19]. Exogenously introduced Hh pathway components in these cells were able to reconstitute Hh pathway response (Fig 3). We observed that N-terminally FLAG-tagged Ci (FLAG-Ci), when expressed alone, did not undergo normal proteolytic processing to form CiR (Fig 3A), and displayed anomalously high *ptc*-luciferase activity even in the absence of Hh stimulation (Fig 3B). However, exogenously-directed expression of core pathway components, Smo, Cos2, Fu, and Su(fu), together with FLAG-Ci, enabled Ci proteolytic processing and reliable Hh-dependent posttranslational modifications as well as *ptc*-luciferase activity (Fig 3A and 3B; for a more detailed summary, see S1 Text).

We furthermore noted that Fu^K33A^, which suppresses response in cl-8 cells (Fig 2), also suppresses response in this system (Fig 3C). Notably, whereas Fu^K33A^ could completely suppress the Hh-stimulated *ptc*-luciferase activity when Smo and Cos2 were co-expressed (Fig 3C), it could not do so when Cos2 expression was omitted (Fig 3C), suggesting that the *trans*-dominant activity of Fu^K33A^ is mediated by Cos2, and consistent with Su(fu)-independent action of Fu. Altogether, these results demonstrate that our reconstitution system mimics the behavior of cells with endogenous Hh pathway components.

### Ci interaction with Su(fu) or Cos2 correlates with Su(fu)- or Cos2-mediated suppression of Ci activity

**Fig 4:**
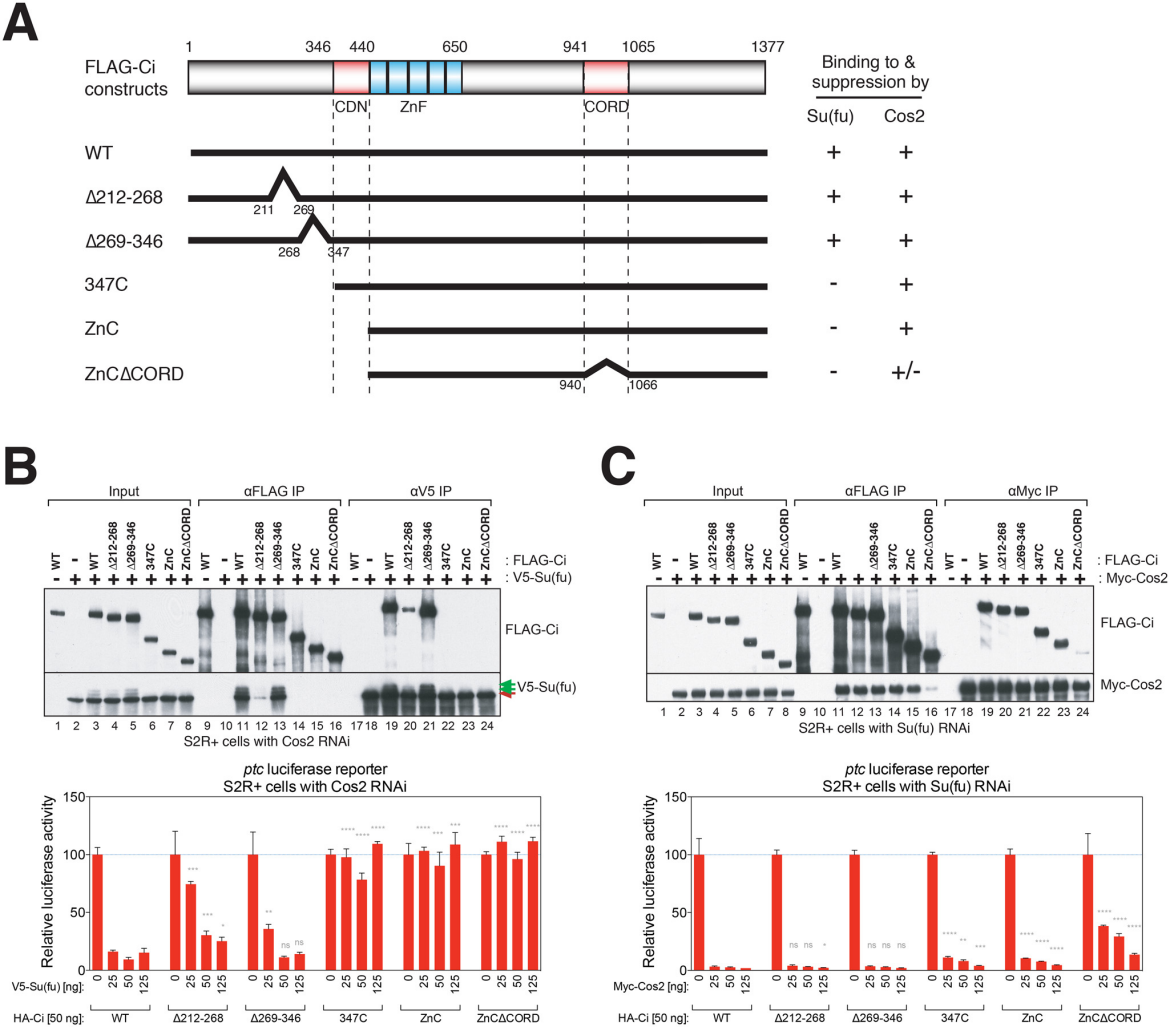
Physical and functional characterization of Su(fu) and Cos2 interactions with Ci. (A) Schematic representation of Ci variants and their interactions with Su(fu) and Cos2. (B) Su(fu) represses Ci activity through binding to a.a. 1–346 of Ci. (Top) S2R+ cells were transfected with indicated expression constructs, and with dsRNA against Cos2 to deplete endogenous Cos2, which could indirectly bring V5-Su(fu) to FLAG-Ci constructs through endogenous Fu. (Bottom) Su(fu) binding is required for Ci suppression by Su(fu). Increasing amounts of V5-Su(fu) (0, 25, 50, 125 ng) and fixed amount of FLAG-Ci constructs (25 ng) were transfected in S2R+ cells with Cos2 RNAi to deplete endogenous Cos2. (C) CDN and CORD mediate most but not all of the Cos2 binding and repression of Ci. (Top) S2R+ cells were transfected with dsRNA against Su(fu) as well as indicated expression constructs, and subjected to either anti-FLAG or anti-Myc IP. (Bottom) Increasing amounts of Myc-Cos2 (0, 25, 50, 125 ng) and fixed amount of FLAG-Ci constructs (25 ng) were expressed in S2R+ cells with Su(fu) RNAi to deplete endogenous Su(fu). A representative experiment from at least three independent experiments is shown. Error bars show mean +/- standard deviation. Statistical significance was measured by Student’s *t*-test: **** (P<0.0001), *** (0.0001<P<0.001), ** (0.001<P<0.01), * (0.01<P<0.05), and ns (not significant, P>0.05). P values in (B) and (C) were calculated for pairs of the same DNA mass of V5-Sufu (B) or Myc-Cos2 (C) construct between WT Ci-expressing cells and a Ci deletion/truncation variant-expressing cells.

As the S2R+ cell reconstitution system depends entirely on exogenously added Ci, we were able to test altered forms of Ci with modifications of Su(fu) binding regions. We constructed several FLAG-Ci variants, deleting previously reported Su(fu)-binding (aa212-268) (CiΔ212–268) [42,43] and Cos2-binding regions (CDN (aa347-440) and CORD (aa942-1065)) (CiΔCDN and CiΔCORD, respectively) [16,63], or either the entire region N-terminal to CDN (Ci347C) or to the zinc-finger domain including CDN (CiZnC [64] and CiZnCΔCORD) (Fig 4A), and analyzed by co-IP their ability to bind to either V5-Su(fu) or Myc-Cos2. We then tested the ability of either Su(fu) or Cos2 to suppress the transcriptional activity of the FLAG-Ci deletion variants in S2R+ cells by measuring *ptc*-luciferase activity. We found that V5-Su(fu) strongly suppressed the transcriptional activity of co-expressed WT Ci and CiΔ269–346 (Fig 4A and 4B). The transcriptional activity of CiΔ212–268, which can weakly bind to V5-Su(fu) (Fig 4A and 4B), was suppressed at higher levels of V5-Su(fu) than that of WT Ci and CiΔ269–346 (Fig 4B). In contrast, V5-Su(fu) even at the highest amounts did not suppress the transcriptional activity of Ci347C, CiZnC, and CiZnCΔCORD (Fig 4B), all of which did not show detectable Su(fu) binding (Fig 4A and 4B).

On the other hand, Myc-Cos2 co-IP’ed CiΔ212–268, CiΔ269–346 and Ci347C, each of which possesses both CDN (aa347-440) and CORD (aa942-1065). Strong binding to Myc-Cos2 persisted with CiZnC, which lacks CDN but retains CORD [64] (Fig 4A and 4C). Moreover, deletion of CORD from CiZnC (CiZnCΔCORD) dramatically decreased but did not completely eliminate detectable Cos2 binding (Fig 4A and 4C). Thus, CDN and CORD are responsible for most but not all Cos2 binding, implying the presence of an additional unidentified Cos2-binding region within Ci; the third, fourth, and fifth zinc fingers of Ci recently were reported to constitute such an additional region [65], and this region indeed is present in our CiZnCΔCORD construct. Myc-Cos2 strongly suppressed the transcriptional activity of all the co-expressed Ci variants except CiZnCΔCORD (Fig 4C). Nevertheless, high amounts of Myc-Cos2 suppressed the transcriptional activity of CiZnCΔCORD as well, consistent with the weak binding between CiZnCΔCORD and Cos2 (Fig 4A and 4C) and with the recently reported interaction of Ci zinc fingers with Cos2 [65]. These results demonstrate that binding of Ci to either Cos2 or Su(fu) in this cellular reconstitution system is associated with suppression of basal Ci transcriptional activity.

### Ci lacking Su(fu)-binding determinants is responsive to Fu and Hh stimulation

**Fig 5:**
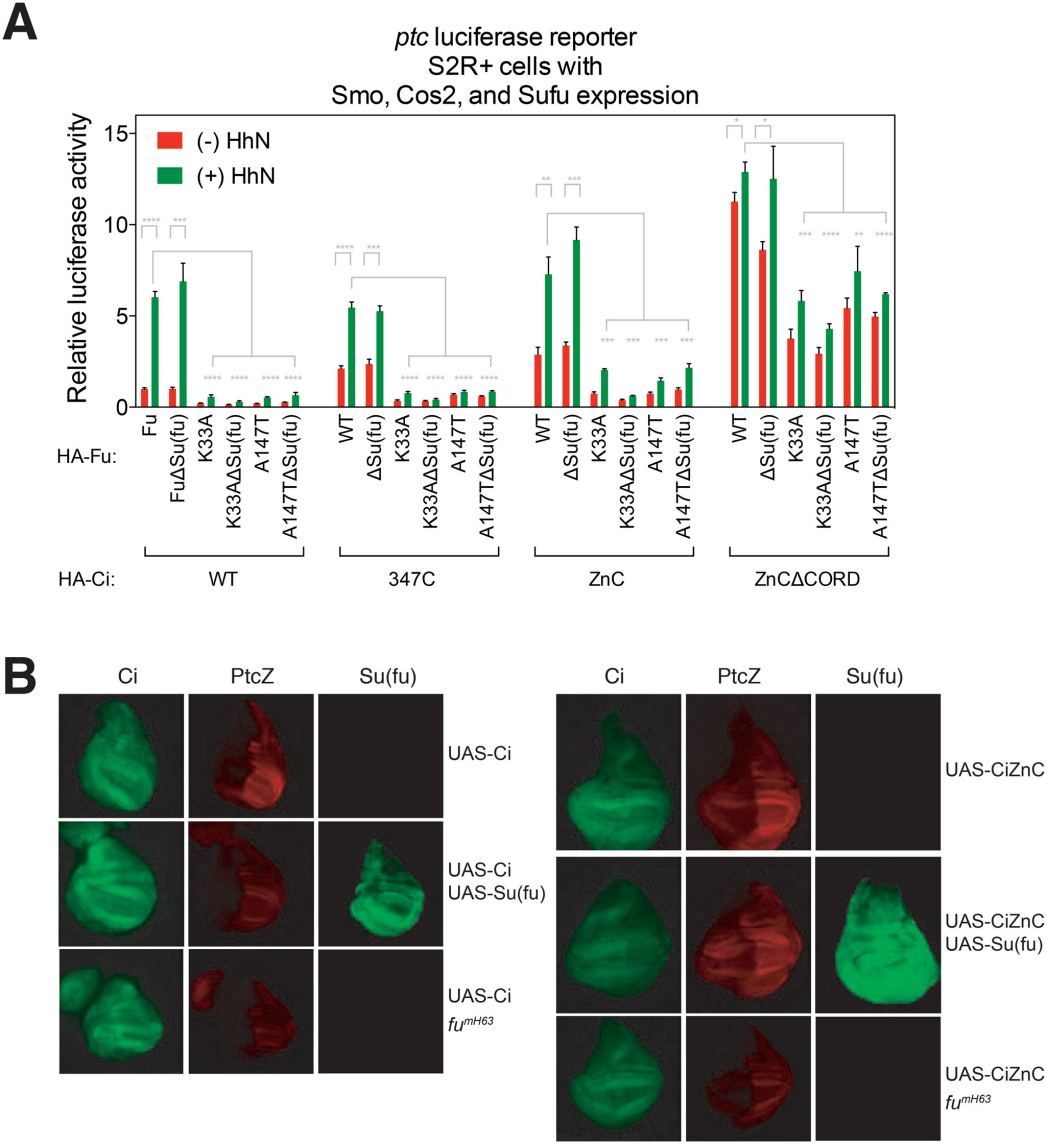
Fu kinase activates Ci independently of Ci suppression by Su(fu). (A) S2R+ cell reconstitution assay to test activity of Ci variants. Exogenous expression of core pathway components, Smo, Cos2, Fu, and Su(fu), with Ci produced a reliable Hh-dependent *ptc*-luciferase activity. (B) Su(fu)-independent activation of Hh-dependent transcriptional targets in the *Drosophila* wing imaginal disc, as monitored by the *ptc-lacZ* reporter. Activities of UAS-Ci or UAS-CiZnC, expressed throughout the wing disc under control of the C765-Gal4 driver, were monitored by immunostaining for β-galactosidase (red) and Ci (green). A representative experiment from at least three independent experiments is shown. Error bars show mean +/- standard deviation. Statistical significance was measured by Student’s *t*-test: **** (P<0.0001), *** (0.0001<P<0.001), ** (0.001<P<0.01), and * (0.01<P<0.05).

To test the role of Su(fu) in Hh stimulation in the cellular reconstitution system we introduced FLAG-Ci variants with and without the ability to interact with Su(fu) along with Su(fu), Smo, Cos2 and measured *ptc*-luciferase activity in response to Hh stimulation. We found that WT Fu activated not only WT Ci but also Ci347C and CiZnC (Fig 5A), but only activated CiZnCΔCORD minimally. The response to Hh stimulation correlated with presence of Cos2 binding determinants present in WT, Ci347C and CiZnC, and mostly absent in CiZnCΔCORD (Fig 4A and 4C) and was independent of determinants for binding of Su(fu) to Ci (Fig 4A and 4B). The dependence of Fu-mediated transcriptional activity on Ci interaction with Cos2 but not Su(fu) is also highlighted by the suppressive effects of the kinase-dead Fu variant Fu^K33A^, which requires the determinants for interaction with Cos2, but not Su(fu) (Fig 5A); a similar effect was noted for Fu^A147T^ (Fig 5A), a variant of Fu encoded by the *fu* allele, *fu* ^*mH63*^, with a lesion in the kinase domain [44].

To test the requirement for Su(fu) in Ci induction *in vivo* we examined the expression of *PtcZ* [40], a Hh-responsive *lacZ*-containing P element insertion into the *Ptc* locus, as regulated by WT Ci or CiZnC. The WT and CiZnC forms of Ci were expressed under control of a Gal4-UAS promoter [43] with a C765-Gal4 driver that is active throughout the wing disc [41]. We noted for both Ci and CiZnC that expression of *PtcZ* was significantly higher in the posterior, where Hh is expressed, thus suggesting responsiveness of both of these Ci constructs to the Hh signal (Fig 5B). Co-expression of Su(fu) under Gal4-UAS control reduced activation of the *PtcZ* target for Ci but not CiZnC, as might be expected since CiZnC lacks Su(fu) binding determinants (Fig 5B). Interestingly, although only Ci-driven (and not CiZnC-driven) expression of PtcZ was suppressed by Su(fu) expression, the level of *PtcZ* expression for both Ci and CiZnC was dramatically reduced in the *fu* ^*mH63*^ mutant (Fig 5B), indicating a dependence of both Ci and CiZnC on activity of Fu. These results indicate that both Ci and CiZnC can be activated by Hh stimulation *in vivo* in a manner depending on activity of Fu; this activation can occur independently of Su(fu), as CiZnC lacks Su(fu) binding sites and its activity is not affected in the absence of Su(fu) function.

### Mammalian Hh signaling does not proceed via general Sufu inactivation

**Fig 6:**
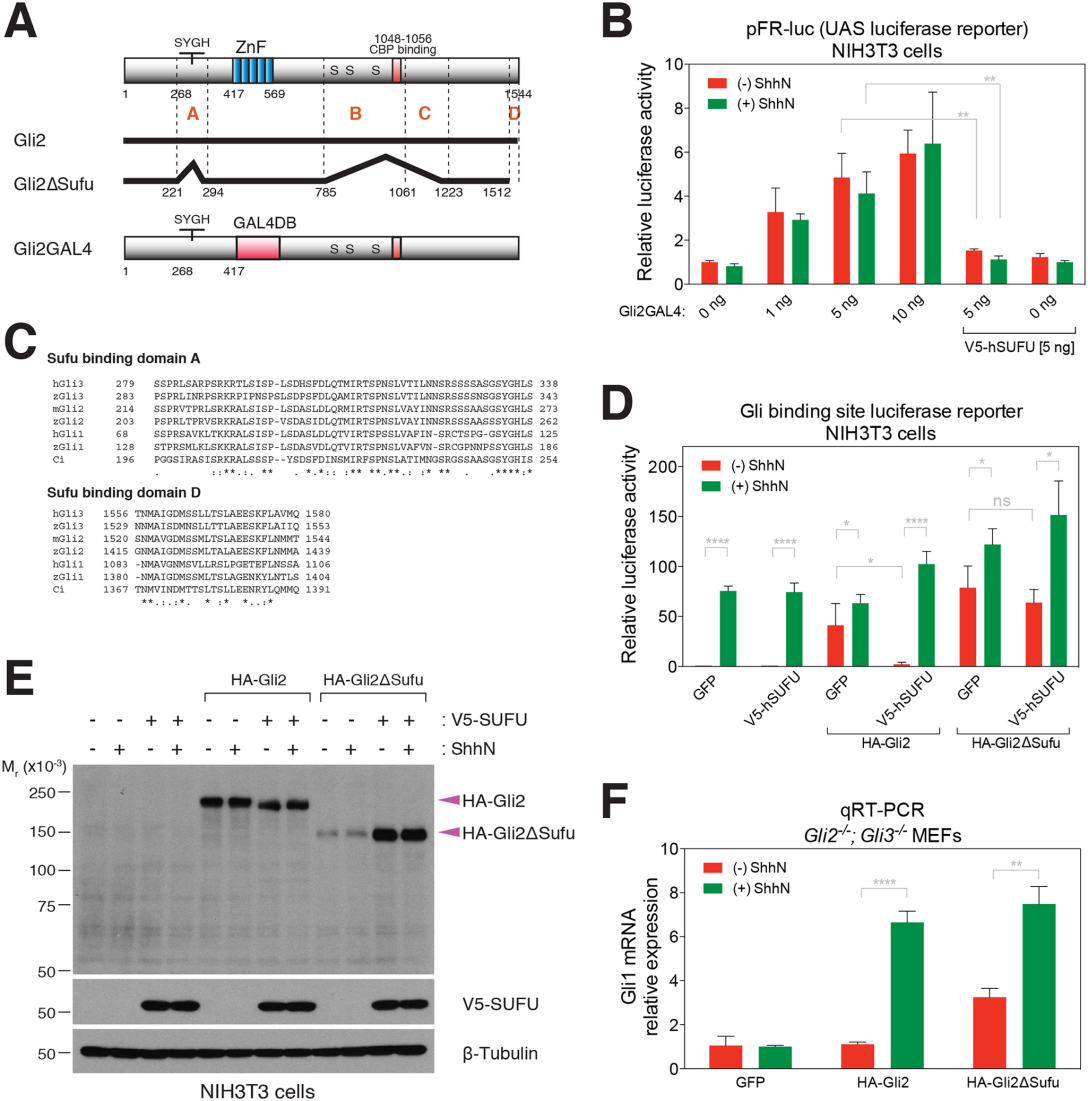
Physical and functional characterization of Gli2 interaction with Sufu. (A) Schematic representation of Gli2 variants used in this study. Gli2 determinants that confer interaction with Sufu is shown in a Gli2 deletion variant Gli2ΔSufu, in which regions A-D indicate Sufu-binding regions (S2 Fig). The SYGH motif is a conserved portion of Sufu binding region A [29]. Gli2GAL4 is a Gli2 variant in which the GAL4 DNA-binding domain replaces the zinc finger domain. (B) NIH3T3 cells transfected for expression of Gli2GAL4 construct with UAS-luciferase reporter (pFR-luc), SV40-*Renilla* and GFP or hSUFU show suppression of luciferase expression by Sufu, but not Shh induction. Cells were stimulated with or without conditioned medium contatining ShhN, the N-terminal signaling domain of the Sonic Hedgehog (Shh) protein. (C) Amino acid sequence alignment of Sufu-binding regions A and D of Gli/Ci proteins. (D) The Gli2ΔSufu variant lacking Sufu-binding regions is resistant to suppression by Sufu overexpression. NIH3T3 cells were transiently transfected with Gli-luc, SV40-*Renilla*, and GFP, Gli2 or Gli2ΔSufu, either alone or in combination with either GFP or hSUFU. (E) Exogenous Sufu expression increases Gli2ΔSufu expression level via a mechanism that is not dependent on direct Gli2/Sufu interaction or ShhN stimulation. HA-Gli2 and HA-Gli2ΔSufu expression in NIH3T3 cells with or without ShhN stimulation was examined by co-transfecting with V5-SUFU construct and Western blot analysis. β-Tubulin is used as a loading control. (F) The Gli2ΔSufu variant lacking Sufu-binding regions has high basal pathway activity but is still inducible upon ShhN-stimulation in *Gli2* ^*-/-*^ *; Gli3* ^*-/-*^ double mutant MEFs. *Gli2* ^*-/-*^ *; Gli3* ^*-/-*^ double mutant MEFs were infected with HA-tagged Gli2 or Gli2ΔSufu coding sequences fused to IRES-GFP in a retroviral expression construct and sorted by GFP intensity. *Gli1* mRNA expression was measured using qRT-PCR to indicate Hh pathway activity. A representative experiment from at least three independent experiments is shown. Error bars show mean +/- standard deviation. Statistical significance was measured by Student’s *t*-test: **** (P<0.0001), *** (0.0001<P<0.001), ** (0.001<P<0.01), * (0.01<P<0.05), and ns (not significant, P>0.05).

Ci response to Hh stimulation in the absence of *Su(fu)* function or response of CiZnC to Hh stimulation in the absence of Sufu-binding determinants is consistent with a nearly undetectable phenotype for the *Su(fu)* mutant in *Drosophila* [34]. The mouse *Sufu* mutant phenotype in contrast is more severe [32,33], and it has been proposed that the role of Sufu in pathway response is more critically important than in *Drosophila* [1,2,4]. To explore possible links between Sufu suppression of transcriptional activity and Hh induction, we considered a mechanism in which mammalian Hh signaling may proceed via inactivation of Sufu function. To examine this possibility, we replaced the Zn fingers of Gli2 with the DNA-binding domain of Gal4 (Gli2GAL4) (Fig 6A). This construct activates transcription of a Gal4 UAS-luciferase reporter (pFR-luc), thus allowing us to examine transcriptional activity independently of endogenous pathway targets such as *Gli1*, which can amplify transcriptional output and thus complicate analysis. We found that Gli2GAL4 produced high activity of the pFR-luc reporter, which was not further induced by ShhN stimulation (Fig 6B). Despite this lack of response to ShhN, pFR-luc reporter activity was suppressed by co-expression of hSUFU (Fig 6B). Similarly, co-expression of SUFU with WT Gli2 in NIH3T3 cells also suppressed basal Hh reporter activity but, in this case, Shh stimulation was able to restore reporter activity (Fig 6D). One possible explanation for the lack of Hh stimulation of Gli2GAL4 activity is that the zinc fingers of the WT Gli2 protein are required for ciliary trafficking, and this ciliary trafficking is disrupted in the Gli2GAL4 protein (data not shown). Our data nevertheless demonstrate that Sufu suppression of transcriptional activity can occur in a manner that is not sensitive to Hh stimulation, indicating that Hh stimulation does not act by incapacitating all Sufu within the cell; the mechanism of Hh signaling thus appears not to involve a general inactivation of Sufu activity.

### Definition of Sufu binding domains within Gli2

Having ruled out general inactivation of Sufu activity as the major mechanism of Hh-dependent transcriptional activation, we considered the role of specific Sufu/Gli protein interactions, as was tested for Su(fu)/Ci in *Drosophila* (see above). Transcriptional output of Hh signaling in mammals is complicated by the presence of three Ci orthologs, the Gli transcription factors [5], and the gene for one of these, *Gli1*, is actually a target of Hh-induced transcriptional activation [10,13]. To determine whether Sufu suppression of Gli activity is required for Hh-induced activation of gene targets, we focused on Gli2, the major mediator of Hh positive transcriptional response [5], and began by mapping determinants in Gli2 that mediate Sufu binding.

A co-immunoprecipitation assay using a series of Gli2 deletions revealed four regions in Gli2 that can each independently bind to Sufu: A (aa 221–294, which contains the well-studied SYGHL motif [29,37,38]), B (aa 785–1061), C (aa 1062–1223) and D (aa 1513–1544) (Fig 6A and 6C, S2 Fig). The presence of any single Sufu binding domain was sufficient to mediate interaction with Sufu, although the B domain binds more weakly than the A, C, or D (S2 Fig), and binding domains A and D are highly conserved among Gli/Ci family proteins (Fig 6C). Simultaneous deletion of all four binding domains abolished interaction with Sufu (S2B Fig), and this construct, Gli2ΔSufu, was used to study the role of Sufu in response to Hh stimulation.

### Sufu-binding regions of Gli2 are not required for response to Hh stimulation

Upon transfection into NIH3T3 cells of a construct for expression of Gli2ΔSufu, we found that its transcriptional activity was similar or slightly higher to that of WT Gli2 (Fig 6D). In contrast to WT Gli2, however, we found that Gli2ΔSufu was resistant to the inhibitory effect of Sufu co-expression (Fig 6D). We further noted that, although Gli2ΔSufu mediated a relatively high-level Hh transcriptional response, Gli2ΔSufu was expressed at a much lower level than WT Gli2, revealing an intrinsically labile nature of Gli2ΔSufu (Fig 6E). Co-expression of exogenous human SUFU, however, restored Gli2ΔSufu expression to a level comparable to that of Gli2 in both ShhN-treated and—untreated cells (Fig 6E). Increasing Sufu expression thus appears to rescue the Gli2ΔSufu instability via a mechanism that does not depend on the direct interaction between Sufu and Gli2, regardless of ShhN stimulation.

To evaluate the role of Gli2/Sufu interaction in pathway stimulation, we wished to test the Hh-inducibility of Gli2ΔSufu; however, NIH3T3 cells contain endogenous Gli2, which is likely to be responsible for at least a portion of the increased reporter expression observed upon ShhN stimulation. We therefore made use of mouse embryonic fibroblasts lacking both *Gli2* and *Gli3* (*Gli2* ^*-/-*^ *; Gli3* ^*-/-*^ MEFs) [50] to develop a reconstitution assay in which expression of Hh pathway transcriptional targets, including endogenous *Gli1*, is dependent upon an exogenously introduced construct for expression of Gli2.

*Gli2* ^*-/-*^ *; Gli3* ^*-/-*^ MEFs were separately transduced either with a retrovirus carrying the Gli2 coding sequence or the Gli2ΔSufu coding sequence, in each case followed by an internal ribosome entry sequence (IRES) and coding sequences for green fluorescent protein (GFP). Positive selection by FACS for GFP expression was used to ensure viral transduction and to eliminate cells in the top 50%, thus ensuring expression of Gli2 at moderate levels that might respond to regulation by Hh stimulation. Using endogenous *Gli1* mRNA levels as a sensitive readout of Hh pathway activity using qRT-PCR, we found that Gli2-expressing cells display Hh-dependent induction of the endogenous *Gli1* transcript (Fig 6F). The Gli2ΔSufu-expressing line in contrast displayed a higher basal *Gli1* mRNA level; this level of expression nevertheless was elevated by ShhN stimulation (Fig 6F). These data suggest that interaction between Sufu and Gli2 contributes to constitutive suppression of targets such as *Gli1*, but also that Hh-stimulated activation of Gli2 may to some degree occur independently of Sufu, as we also noted in the *Drosophila* analysis of these proteins.

### Distinct ciliary trafficking mechanisms for Gli2 and Sufu

**Fig 7:**
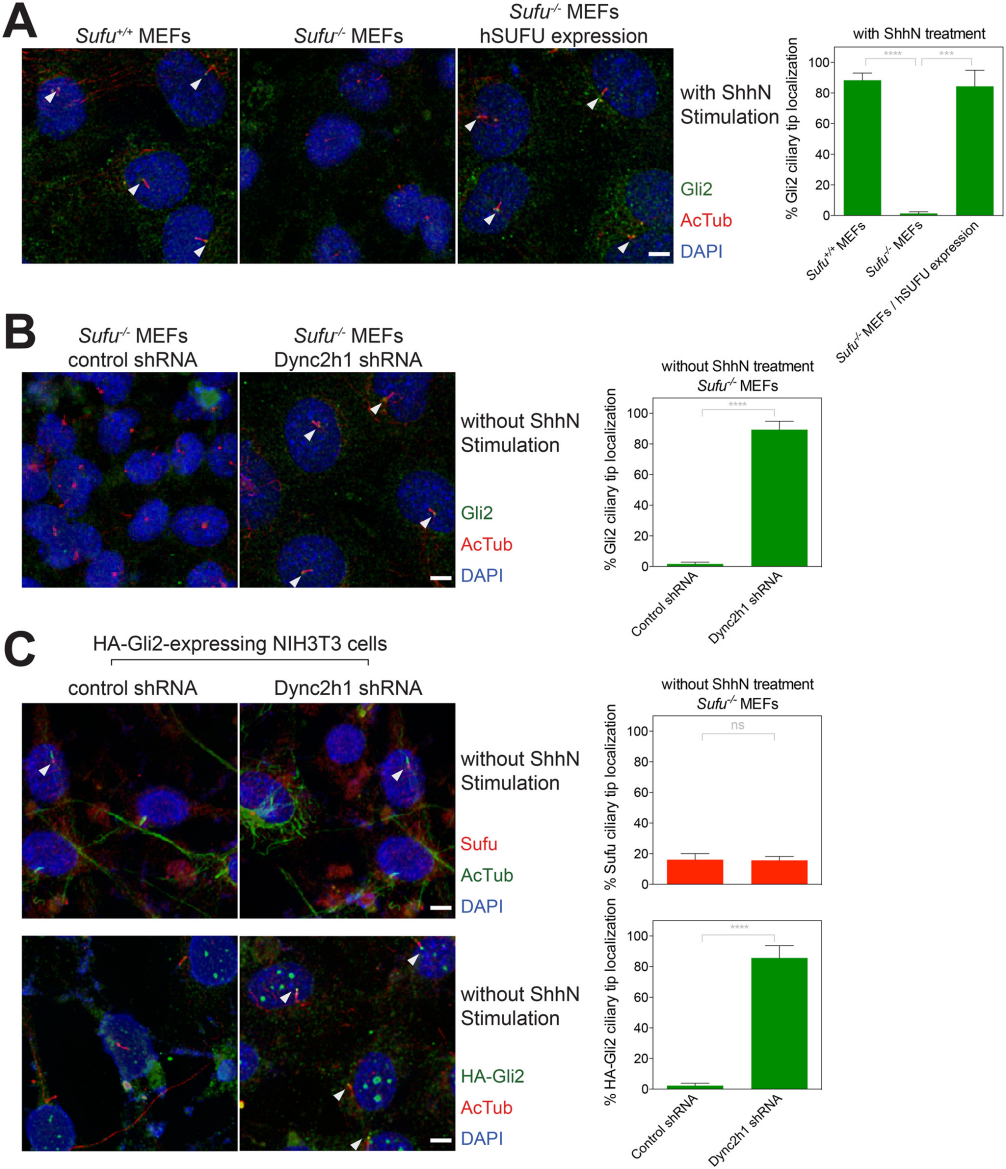
Ciliary trafficking of Gli2 in the absence of Sufu function. Ciliary localization of endogenous Gli2 is undetectable in *Sufu* ^*-/-*^ MEFs even though Gli2 shuttles in and out of cilia. (A) Immunofluorescence staining of *Sufu* ^*-/-*^ MEFs and *Sufu* ^*-/-*^ MEFs rescued with human SUFU (hSUFU) for Gli2 (green) and acetylated Tubulin (AcTub, red) and DAPI (blue). (B) Immunofluorescence staining of *Sufu* ^*-/-*^ MEFs stably transfected for expression of Dync2hc or control shRNA with antibodies against Gli2 (green) and AcTub (red). (C) Unlike Gli2, a block of retrograde transport in primary cilia by *Dync2hc* shRNA does not increase ciliary accumulation of Sufu. NIH3T3 cells stably expressing HA-Gli2 with shRNA targeting either Dync2h1 or control were stained with antibodies against AcTub (green) and HA or Sufu (red). Arrowheads denote ciliary tip localization. A representative experiment from at least three independent experiments is shown. Ciliary tip localization of Gli2, Sufu, and HA-Gli2 was scored from more than 50 cells per each replicate and plotted in graphs right to immunofluorescence images. Error bars show mean +/- standard deviation. Statistical significance was measured by Student’s *t*-test: **** (P<0.0001), *** (0.0001<P<0.001), and ns (not significant, P>0.05).

To investigate the possible role of ciliary trafficking in Sufu regulation of Gli2 responsiveness to ShhN stimulation, we examined the localization of Gli2 and Smo in cells lacking function of *Sufu*, or of the retrograde ciliary trafficking motor, Dynein2. We and others have previously noted that Gli2 accumulation at the tip of the primary cilium increases upon Hh stimulation [22,39,48]. This accumulation does not require Hh-induced *de novo* initiation of ciliary transport, as Gli2 and Smo appear to traffic through the cilium constitutively, even in the absence of Hh stimulation [48,66]. To examine the potential role of Sufu in ciliary trafficking of Gli2, we examined *Sufu* ^*-/-*^ fibroblasts, and found that, although pathway activity is aberrantly high, ShhN-induced accumulation of endogenous Gli2 at the ciliary tip was not detectable in these cells; such accumulation of Gli2 at the ciliary tip was restored in these cells by retroviral transduction for expression of Sufu (Fig 7A). These results could be due to changes in ciliary trafficking or to difficulties in detection of Gli2 due to reduced levels in cells lacking *Sufu* function [35,39,67]. To further explore the possibility of a defect in ciliary trafficking, we transduced *Sufu* ^*-/-*^ MEFs with a retrovirus for expression of an shRNA targeting mRNA for the Dynein2 heavy chain (Dync2h1), and noted that Gli2 accumulated in cilia, even without Hh stimulation (Fig 7B). We noted, in contrast, that the low levels of Sufu present at the ciliary tip in unstimulated cells were not increased by targeting of Dynein2 (Fig 7C). These results indicate that mechanisms for ciliary exit of Sufu and Gli2 differ, consistent with a previous report indicating distinct mechanisms of trafficking [68]. In addition, the constitutive trafficking of Gli2 through the primary cilium occurs in *Sufu* ^*-/-*^ cells in the absence of Hh stimulation, as previously reported for wild-type cells [48]. The continued trafficking of Gli2 through the cilium in the absence of *Sufu* function suggests the possibility that the cilium could still play a role in pathway activation by Hh stimulation, even when Sufu is not present.

### Loss of *Gli1* function partially rescues the *Sufu* mutant phenotype

Our cultured cell data suggest: 1) that Hh signaling does not induce Gli-mediated transcriptional activity by general inactivation of Sufu (Fig 6B); 2) that ciliary trafficking of Gli2, required for its activation, occurs by a distinct mechanism from trafficking of Sufu (Fig 7); and 3) that Gli2 activity can be regulated by ShhN signaling independently of Sufu suppression (Fig 6D and 6F). A similar independence from Su(fu) function for Ci activation was noted in *Drosophila* (see above). However, although the phenotype of *Su(fu)* homozygous mutants in *Drosophila* is minimal, loss of *Sufu* function is embryonic lethal in mice with a phenotype resembling that of *Ptch* ^*-/-*^ mice [32,33]. This suggests that the Hh pathway in *Sufu* ^*-/-*^ mice is activated in a ligand-independent manner to a much greater extent than in *Drosophila*.

**Fig 8:**
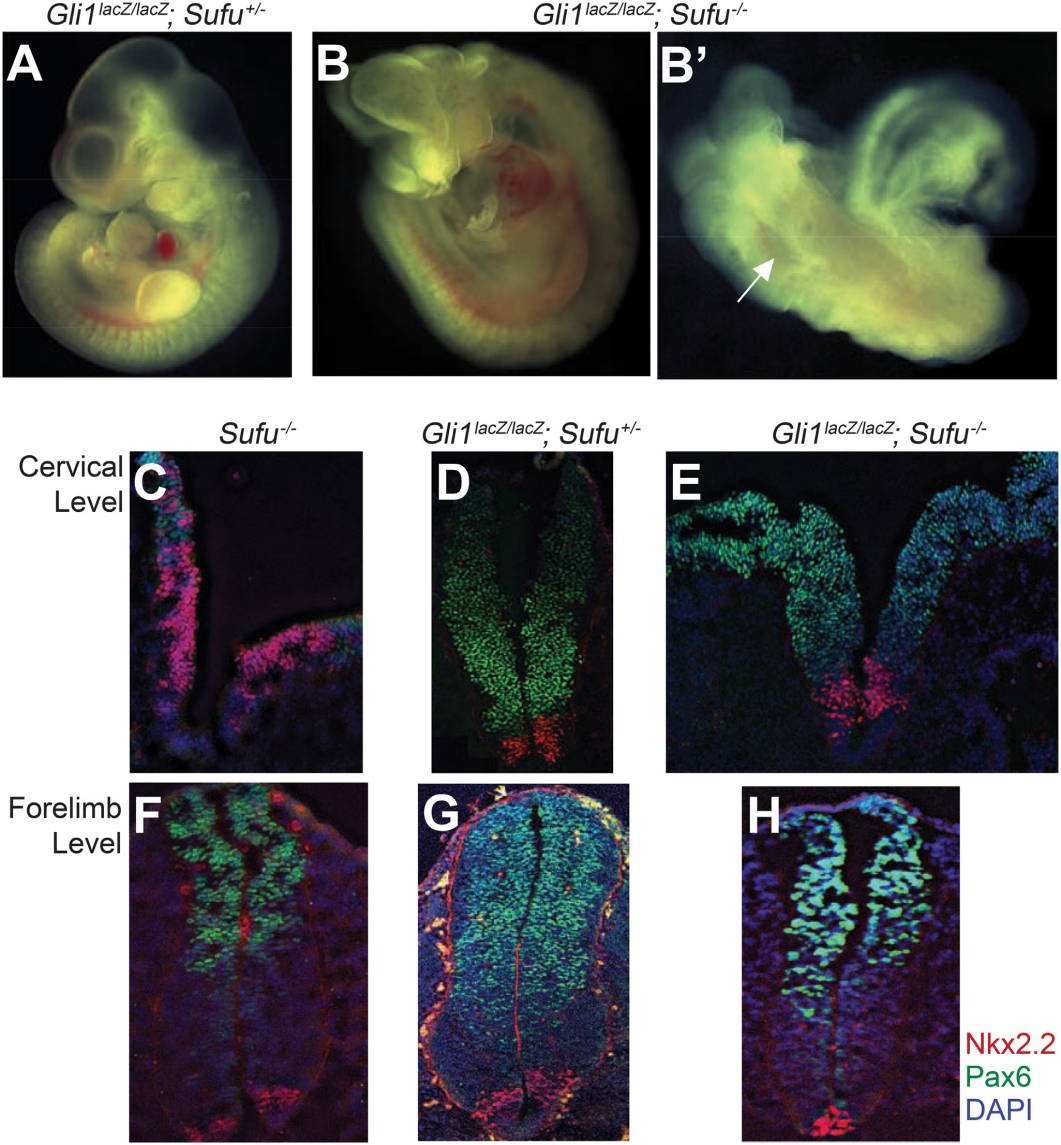
Loss of *Gli1* partially rescues the Sufu mutant phenotype. (A-B’) *Gli1* ^*lacZ/lacZ*^; *Sufu* ^*+/-*^ (A) and *Gli1* ^*lacZ/lacZ*^; *Sufu* ^*-/-*^ (B and B’) E10.5 embryos. *Gli1* ^*lacZ/lacZ*^; *Sufu* ^*-/-*^ embryos survive beyond E10.5, whereas *Sufu* ^-/-^ embryos die around E9.5. *Gli1* ^*lacZ/lacZ*^; *Sufu* ^*-/-*^ shows an open neural tube phenotype (B’) (indicated by a white arrow). (C-E) Transverse sections of the neural tube at the cervical level of E9.5 *Sufu*-/- (C), E10.5 *Gli1* ^*lacZ/lacZ*^; *Sufu* ^*+/-*^ (D), and *Gli1* ^*lacZ/lacZ*^; *Sufu* ^*-/-*^ embryos (E) immunostained for markers of neural fate (Nkx2.2: red, Pax6: green, DAPI: blue). In *Sufu* mutants that survive to E9.5, the ventral spinal cord marker Nkx2.2 expands dorsally (C). However, loss of Gli1 function from *Sufu* ^-/-^ mutant greatly reduced the expression of Nkx2.2 and restored the expression of Pax6 (E). (F-H) Neural tube sections at the level of the forelimb were immunostained for markers of neural fate (Nkx2.2: red, Pax6: green, and blue: DAPI). Embryos were *Sufu* ^-/-^ (E9.5) (F), *Gli1* ^*lacZ/lacZ*^; *Sufu* ^*+/-*^ (E10.5) (G), and *Gli1* ^*lacZ/lacZ*^; *Sufu* ^*-/-*^ (E10.5) (H). Note that expression of Nkx2.2 at the level of the forelimb does not expand to more dorsal regions in the *Sufu* ^-/-^ mutant. The expression of Nkx2.2 in *Gli1* ^*lacZ/lacZ*^; *Sufu* ^*-/-*^ embryos is reduced compared to that in *Gli1* ^*lacZ/lacZ*^; *Sufu* ^*+/-*^ embryos. A representative experiment from at least three independent experiments is shown.

One possible explanation for this difference is that the levels of the Gli proteins, which separately execute transcriptional activation and repression functions, are differentially affected by the absence of Sufu [39,69]. As most of our studies of Sufu-mediated transcriptional activation thus far have focused on Gli2, it is possible that pathway activation is associated with activity of Gli1, which although not essential for development or fertility [12], is a potent transcriptional activator [5] and could be contributing to pathway activity seen in the absence of *Sufu* function. Consistent with this idea, it has been reported that knockdown of *Gli1* expression inhibits Hh pathway activity in *Sufu* ^*-/-*^ MEFs [39]. In order to examine the contribution of *Gli1* to the *Sufu* mutant phenotype, we intercrossed *Gli1* ^*lacZ/lacZ*^ *; Sufu* ^*+/-*^ mice. Of 44 embryos recovered at E10.5, 10 were *Gli1* ^*lacZ/lacZ*^ *; Sufu* ^*-/-*^ indicating no decrement in survival. By E11.5, however, only 1 of 20 embryos recovered was of the genotype *Gli1* ^*lacZ/lacZ*^ *; Sufu* ^*-/-*^, indicating that these embryos mostly die between E10.5 and E11.5. *Gli1* ^*lacZ/lacZ*^ *; Sufu* ^*-/-*^ mutants thus survive 1 day longer as compared to *Sufu* ^*-/-*^ embryos, which die around E9.5 [32,33]. *Gli1* ^*lacZ/lacZ*^ *; Sufu* ^*-/-*^ embryos at E10.5 displayed open neural tubes, exencephaly and defects of limb bud formation (Fig 8A, 8B and 8B’).

To further examine the effect of *Gli1* loss on the *Sufu* mutant phenotype, we analyzed Hh-regulated markers of dorsal/ventral (DV) patterning of the neural tube. Although the expression of such markers is nearly normal at more caudal levels of the neural tube, such as at the level of the forelimb (Fig 8H), the pattern is strikingly disrupted at cervical levels (Fig 8E), where the neural tube remains open (Fig 8B’). Shh normally induces formation of ventral neural progenitors from neural plate cells; expression of *Nkx2*.*2*, which encodes a homeodomain transcription factor, thus marks progenitors of the V3 interneurons, which normally develop adjacent to Shh-expressing cells of the floor plate and thus represent a high-threshold Hh-induced cell fate [70]. Expression of the homeodomain gene *Pax6*, in contrast normally occurs laterally within the developing neural tube and is suppressed by Shh signaling [70]. We noted, as reported previously [32,33], that expression of these genes is dramatically altered in *Sufu-/-* embryos at the cervical level, with expansion of Nkx2.2 expression into lateral and dorsal regions, and a corresponding loss of *Pax6* expression (Fig 8C). We found that additional loss of *Gli1* (*Gli1* ^*lacZ/lacZ*^ *; Sufu* ^*-/-*^ embryos) restored expression of Nkx2.2 and Pax6 to their normal domains, producing a pattern like that of wild-type or *Gli1* ^*lacZ/lacZ*^ *; Sufu* ^*+/-*^ embryos (Fig 8D and 8E).

These data suggest that a great deal of the mispatterning in *Sufu* ^*-/-*^ embryos, at least at rostral levels, depends on the function of *Gli1* and thus, *Gli1; Sufu* double mutants do not show as severe a developmental phenotype as the *Sufu* ^*-/-*^ mutant. These data furthermore suggest that neural tube patterning can occur independently of Sufu when Gli1 is absent, presumably through Hh regulation of Gli2 and Gli3 and consistent with our cultured cell data (Fig 6) and with Hh regulation of Ci in the absence of Su(fu) influence in *Drosophila* (Fig 5).

### A positive role for Sufu in Hh signaling

**Fig 9:**
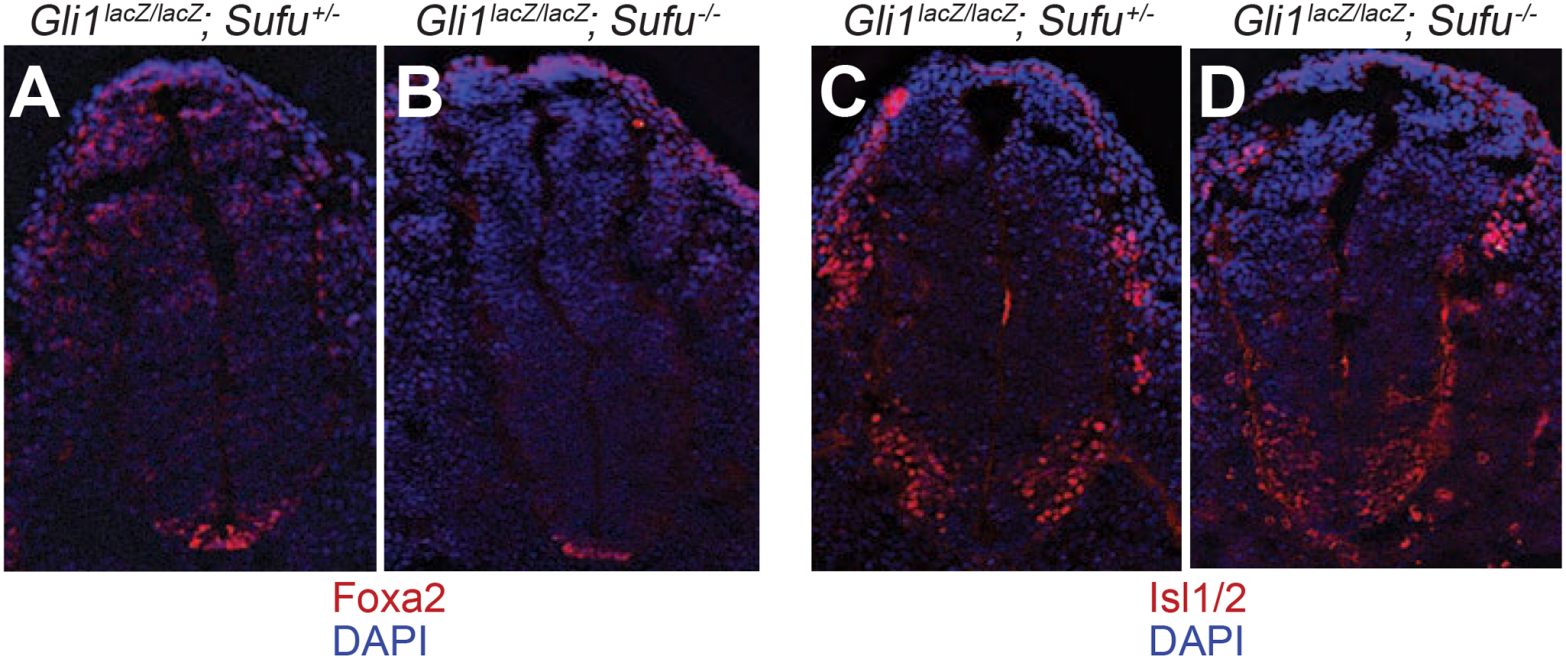
A positive role for Sufu in Hh signaling in neural tube patterning at forelimb level. Neural tube sections from E10.5 embryos at the level of the forelimb were immunostained for markers of neural fate (red: Foxa2 (A and B) or Isl1/2 (C and D), blue: DAPI) in *Gli1* ^*lacZ/lacZ*^; *Sufu* ^*+/-*^ (A and C), and *Gli1* ^*lacZ/lacZ*^; *Sufu* ^*-/-*^ embryos (B and D) Note that in the *Gli1* ^*-/-*^ background, floor plate formation (marked by expression of Foxa2) is reduced by the absence of *Sufu* function. A representative experiment from at least three independent experiments is shown.

Interestingly, our evidence also suggests the possibility that Sufu activity in the mouse neural tube at the level of the forelimb may contribute positively to Hh pathway response, as would be consistent with a recent report based on cultured cell assays [39]. We thus find that expression of the floor plate marker, Foxa2, in mice lacking *Gli1* function but retaining a single wild-type allele of *Sufu* (*Gli1* ^*lacZ/lacZ*^ *; Sufu* ^+/-)^ appears normal (Fig 9A and 9B). Upon complete loss of *Sufu* function, however, Foxa2 expression is actually reduced (*Gli1* ^*lacZ/lacZ*^ *; Sufu* ^*-/-*^ *)* (Fig 9C and 9D); in addition, a corresponding ventral shift in expression of more lateral markers such as *Nkx2*.*2* and *Isl1/2* was noted with the complete loss of *Sufu* function in the *Gli1* ^*lacZ/lacZ*^ background (Fig 8H). An alternative interpretation of our result, however, could be that failure of floor plate development may be caused by developmental delay, or by the lack of downregulation of Hh response that is required for its differentiation [71].

## Discussion

The primary amino acid sequence of *Drosophila* and mammalian Sufu shows 38% identity, higher than that of *Ptc*/*Ptch*, Smo, or Ci/Gli. Despite this conservation, genetic analysis of Sufu function suggests quite different roles in these organisms, with a minimal mutant phenotype in *Drosophila* and an embryonic lethal phenotype and ligand-independent pathway activation in homozygous mouse mutants. Because of this apparent contradiction, we undertook a more comprehensive comparison of the role of Sufu in these distinct organisms.

### Phosphorylation of *Drosophila* Su(fu) is not functionally important

A prevailing model for Sufu function in *Drosophila* is that Fu kinase increases Ci transcriptional capability by phosphorylating and inactivating Su(fu) [1,57]. This model is based on the observations that Hh stimulation induces phosphorylation of Su(fu), which decreases with loss of Fu [19,56], and that Fu can physically interact with Su(fu) [54]. To examine this model we identified phosphoresidues within Su(fu) and functionally tested Su(fu) proteins in which these residues were altered. We found no difference in the function of these altered Su(fu) proteins. We also defined a 30 amino acid region of Fu that is required for interaction with Su(fu), and found that this region is not required for Fu function in Hh signaling; given the evident lack of importance of Su(fu) phosphorylation, we suggest that the Hh-induced increase in Su(fu) phosphorylation may be due to changes in activity or complex formation by kinases whose primary function is not the modulation of Su(fu) activity.

### Ci inducibility without Su(fu) interaction

We also defined the region of Ci that interacts with Sufu and tested whether Hh signaling can induce transcriptional activity of Ci proteins lacking this interaction. We found, using a cultured cell reconstitution assay that requires exogenously introduced Ci and other pathway components, that expression of target genes can be induced even under conditions in which Ci-Su(fu) interaction is abolished. This phenomenon was also observed *in vivo* in transgenic flies expressing a Ci variant lacking Su(fu) interaction. Interestingly, Hh stimulation of Ci-mediated transcription *in vivo* required Fu activity, even with the Ci variant lacking Su(fu) interaction. Taken together, these results show that Su(fu) is not required for Hh-dependent stimulation of Ci transcriptional activity.

### Inducibility of Gli2 activity without Sufu interaction

In our analysis of mammalian Sufu function we first excluded the general inactivation of Sufu as a mechanism for pathway activation by demonstrating that a Gli2 protein with a GAL4 DNA-binding domain, although it is an artificial synthetic protein, can be inactivated by Sufu, even when stimulated by ShhN. We then focused on Gli2, the major transcriptional effector of Hh signaling, and defined four regions of Gli2 that interact with Sufu, including the extensively characterized region containing the converved SYGHL motif within the amino-terminal portion of all three Gli proteins [29,37,38]. Using a cell line that lacks *Gli2* and *Gli3* functions, we then established a reconstitution assay, which requires exogenously introduced Gli2 for pathway response, and used this assay to demonstrate that a Gli2 variant lacking Sufu-interacting regions is still capable of some response to Shh stimulation. We thus observe a similar ability of Ci in *Drosophila* and Gli2 in mammalian cells to respond to some extent to Hh signaling in the absence of interaction with Sufu. Consistent with a Sufu-independent mode of Gli2 regulation, we noted that Sufu and Gli2 ciliary trafficking occurs by distinct mechanisms, and that Gli2 traffics through the cilium in the presence or absence of Sufu function.

### Role of *Gli1* in the mammalian *Sufu* phenotype

To account for the striking differences in impact of *Su(fu)* mutations in *Drosophila* as compared to the mouse, we considered the possibility that multiple Gli proteins in the mouse may be relevant. In particular, we examined the possibility that Gli1 may be providing the transcriptional drive that contributes to inappropriate pathway activity in *Sufu* ^*-/-*^ mutants. We verified this possibility by noting that the *Sufu* ^*-/-*^ embryonic phenotype is significantly suppressed by the additional loss of *Gli1* function, and that neural tube patterning at the cervical level appears nearly normal in these *Gli1* ^*lacZ/lacZ*^; *Sufu* ^*-/-*^ double mutants. Thus, although Sufu clearly plays a critical role in Gli2 regulation, our evidence in a *Gli1* ^*-/-*^ background indicates that some degree of Hh-triggered transcriptional activation via Gli2 can occur independently of Sufu.

### Role of Sufu in increasing the dynamic range of Hh-induced transcriptional output

Sufu in addition has an important role in increasing the dynamic range of transcriptional activation by Hh signaling. As previously established, this occurs through its suppression of Gli2 activity in the unstimulated state and through increased efficiency of Gli3 processing to produce Gli3 repressor [36,67,69,72]. A third mechanism previously proposed to increase dynamic range is that Sufu stabilization of Gli proteins may provide a greater capacity for transcriptional activation once stimulation occurs [39]. This was suggested previously by increased levels of Hh-induced pathway activation in cultured cells expressing Sufu [39]. Our studies are consistent with the operation of this proposed mechanism in the embryo, as the highest levels of pathway activity, represented by floor plate induction marked by Foxa2 in the developing neural tube, appear to be favored by Sufu expression.

One additional mechanism by which Sufu may increase the dynamic range of Hh-induced transcriptional output is through control of a Gli1-mediated positive feedback loop, which could augment or amplify initial transcriptional responses mediated by Gli2. The direct role of Sufu activity in restraining this feedback loop in the embryo was revealed in our studies by suppression of the *Sufu-/-* embryonic patterning phenotype upon additional loss of *Gli1* function. In the wild-type embryo, this loop would be activated only upon Hh stimulation. This critical element of positive feedback is absent in *Drosophila*, as Ci does not positively autoregulate its own transcription; the absence of positive feedback could potentially account for the relatively minor effects of *Su(fu)* loss in *Drosophila*.

## Supporting Information

S1 Fig Poor conservation of the dSu(fu) region containing four phosphorylated serine residues. Multiple sequence alignment of Sufu family proteins by ClustalW. Red dots indicate four phosphorylation sites of dSu(fu) identified by mass spectrometry analysis (Fig 1B). Sequence identity, similarity, and conservation of hydrophilicity/hydrophobicity are indicated by asterisk, colon and period, respectively. dSu(fu); *Drosophila melanogaster*. mSufu; *Mus musculus*. hSufu; *Homo sapiens*. xSufu; *Xenopus laevis*. zSufu; Danio rerio.
(TIF)

S2 Fig Identification of the regions of Gli2 responsible for binding to Sufu. (A) Structures of deletion constructs of Gli2. A, B, C, and D each denote regions capable of independently binding Sufu. (B) Binding interactions between Gli2 and Sufu. HEK293F cells were transiently co-transfected either with HA-tagged mouse Gli2 deletion constructs (full-length, A, B, C, D, and Gli2ΔSufu) or with V5-tagged Sufu constructs (full-length human SUFU, N-terminal half (aa1-261) of human SUFU, C-terminal half (aa262-484) of human SUFU, and full-length *Drosophila* Su(fu) (dSu(fu))). The cell lysates containing HA-tagged Gli2 constructs were mixed with those containing each form of V5-tagged Sufu construct and analyzed by co-immunoprecipitation analysis with anti-HA matrix. WB: Western blot.
(TIF)

S1 Text Reconstitution of Hh Signaling in *Drosophila* S2R+ Cells. (PDF)
