# Supplementary figures and images for: A Comparison of Ci/Gli Activity as Regulated by Sufu in Drosophila and Mammalian Hedgehog Response
Source: PLoS One. 2015 Aug 13;10(8):e0135804. doi: 10.1371/journal.pone.0135804 (PMC4536226; doi:10.1371/journal.pone.0135804)

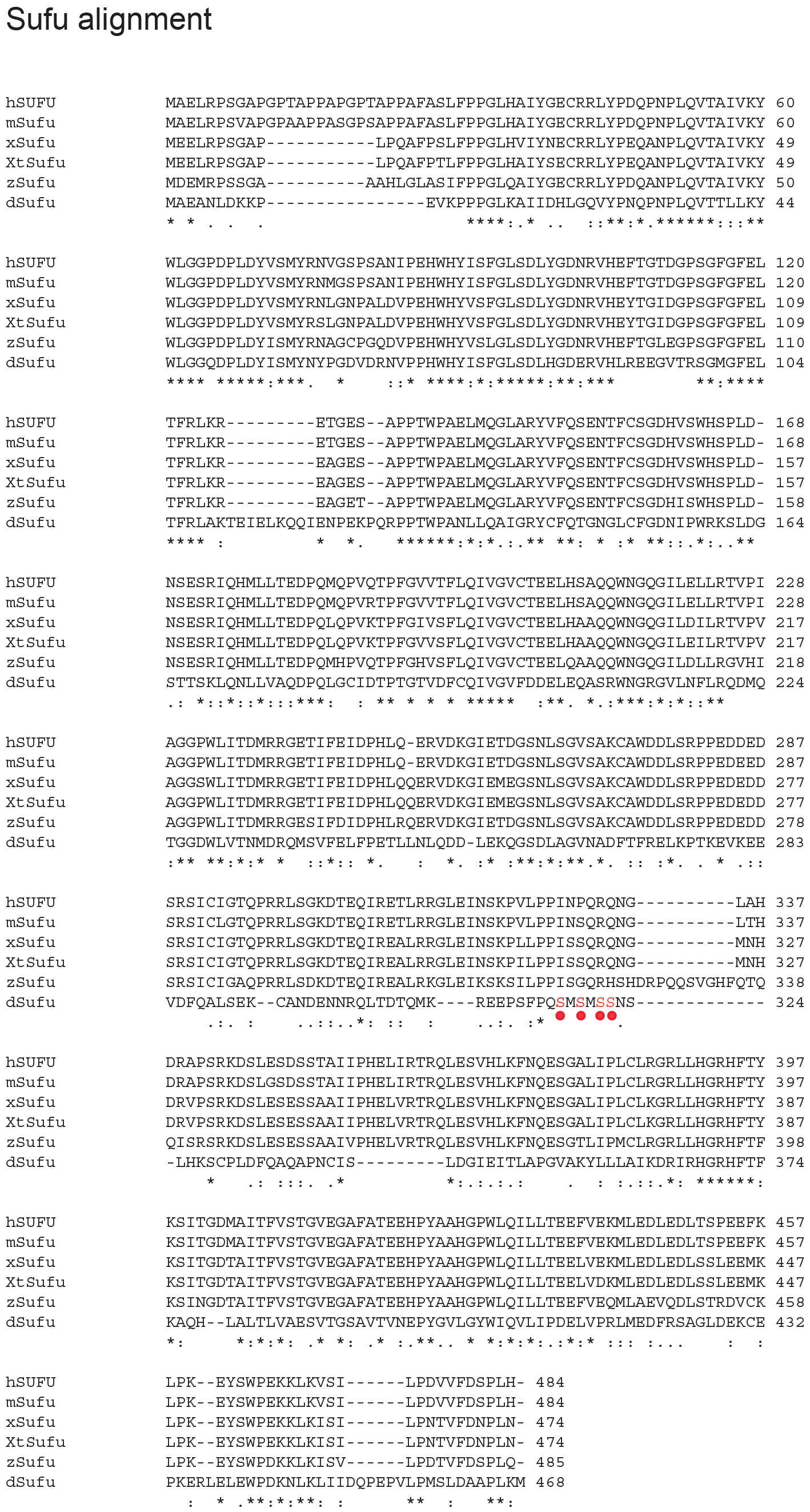

Supplement: S1 Fig — Multiple sequence alignment of Sufu family proteins by ClustalW. Red dots indicate four phosphorylation sites of dSu(fu) identified by mass spectrometry analysis (Fig 1B). Sequence identity, similarity, and conservation of hydrophilicity/hydrophobicity are indicated by asterisk, colon and period, respectively. dSu(fu); Drosophila melanogaster. mSufu; Mus musculus. hSufu; Homo sapiens. xSufu; Xenopus laevis. zSufu; Danio rerio. (TIF) [file pone.0135804.s001.tif]

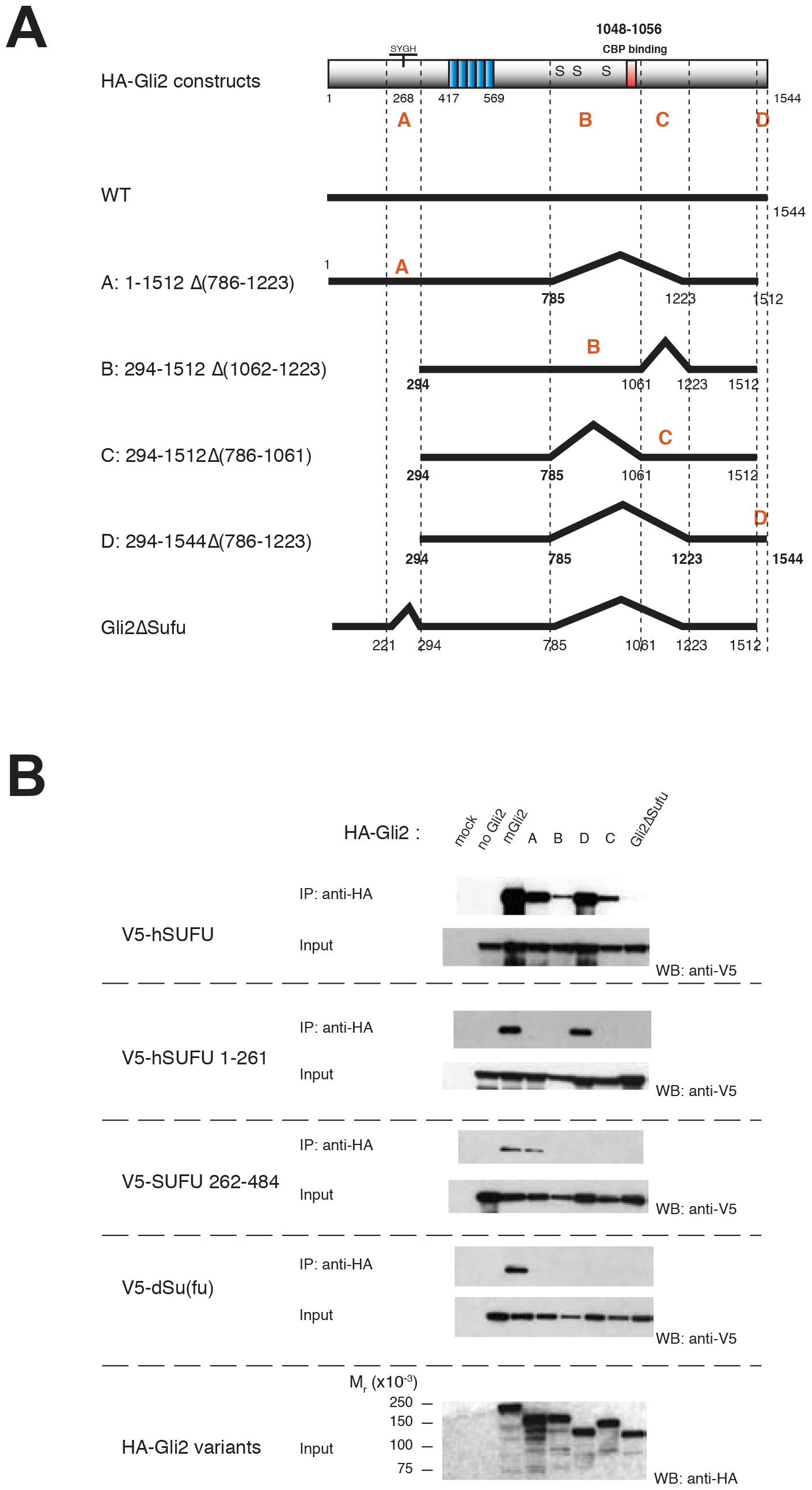

Supplement: S2 Fig — (A) Structures of deletion constructs of Gli2. A, B, C, and D each denote regions capable of independently binding Sufu. (B) Binding interactions between Gli2 and Sufu. HEK293F cells were transiently co-transfected either with HA-tagged mouse Gli2 deletion constructs (full-length, A, B, C, D, and Gli2ΔSufu) or with V5-tagged Sufu constructs (full-length human SUFU, N-terminal half (aa1-261) of human SUFU, C-terminal half (aa262-484) of human SUFU, and full-length Drosophila Su(fu) (dSu(fu))). The cell lysates containing HA-tagged Gli2 constructs were mixed with those containing each form of V5-tagged Sufu construct and analyzed by co-immunoprecipitation analysis with anti-HA matrix. WB: Western blot. (TIF) [file pone.0135804.s002.tif]
