# Supplementary material for: A Comparison of Ci/Gli Activity as Regulated by Sufu in Drosophila and Mammalian Hedgehog Response
Source: PLoS One. 2015 Aug 13;10(8):e0135804. doi: 10.1371/journal.pone.0135804 (PMC4536226; doi:10.1371/journal.pone.0135804)
Supplement: S1 Text — (PDF) [file pone.0135804.s003.pdf]

**S1 Text. Reconstitution of Hh Signaling in *Drosophila* S2R+ Cells**

Since S2R+ cells do not express endogenous Ci, but display all other Hh-induced posttranslational modifications of pathway components, we attempted to reconstitute Hh signaling by exogenously introducing Ci. We observed N-terminally FLAG-tagged Ci (FLAG-Ci), when expressed alone, did not display its CiR form (FLAG-CiR) in Western blot analysis (Fig. 3A, lanes 3 and 4). Co-expression of N-terminally Myc-tagged Cos2 (Myc-Cos2), however, caused FLAG-Ci to undergo the proteolysis process to form FLAG-CiR (Fig. 3A, lanes 7 and 8), suggesting that if sufficient Cos2 is supplied, it can utilize endogenous ancillary factors to promote CiR formation. Whereas co-expression of either N-terminally Myc-tagged Fu (Myc-Fu), V5-tagged Su(fu) (V5-Su(fu), or both along with Myc-Cos2 did not alter the pattern of CiR formation (Fig. 3A, lanes 19-22 and 31-32), co-expression of C-terminally HA-tagged Smo (Smo-HA) with Myc-Cos2 blocked CiR formation and accumulated full-length FLAG-Ci in response to HhN stimulation (Fig. 3A, lanes 13-18, 25-30 and 33-34), consistent with the critical role of Smo-Cos2 interaction to block CiR formation.

We also noted that other exogenously expressed components displayed their own normal posttranslational modifications corresponding to those of endogenous proteins [1-3]. First, Smo-HA became phosphorylated and accumulated in response to HhN stimualation, and co-expression of Myc-Cos2 enhanced the Smo-HA accumulation (Fig. 3A, lanes 5-6, 13-18, 25-30 and 33-34). Second, phosphorylation of Myc-Fu (Fig. 3A, lanes 9-10, 15-16, 19-20, 23-26 and 29-34) and V5-Su(fu) (Fig 3A, lanes 11-12, 17-18, 21-24 and 27-34) increased with HhN stimulation when Smo-HA and Myc-Cos2 were co-expressed. Third, the expression level of Myc-Fu was enhanced with coexpressed Myc-Cos2 (Fig. 3A, lanes 9-10, 15-16, 19-20, 23-26, and 29-34).

We examined whether Ci transcriptional activity correlated with these posttranslational modifications of exogenous pathway components by measuring *ptc*-luciferase activity (Fig. 3B). Expression of FLAG-Ci either alone or with Smo-HA, by which most FLAG-Ci proteins assumed their full-length form (Fig. 3A), resulted in high *ptc*-luciferase activity without HhN stimulation, which was

enhanced only 4-5 fold by HhN stimulation (Fig. 3B). On the contrary, co-expression of Myc-Cos2 without Smo-HA, which promoted formation of FLAG-CiR (Fig. 3A), suppressed *ptc*-luciferase activity both with and without HhN stimulation (Fig. 3B). Co-expressing Smo-HA and Myc-Cos2 with HhN stimulation, which blocked FLAG-CiR formation (Fig. 3A), reversed the Myc-Cos2 effect on pathway suppression (Fig. 3B). Although V5-Su(fu) expression did not affect CiR formation (Fig. 3A), it caused pathway suppression when expressed only with FLAG-Ci (Fig. 3B), consistent with its pathway-suppressing role. Co-expression of Myc-Fu and Smo-HA with or without Myc-Cos2 reversed the Su(fu) effect (Fig. 3B), reflecting the antagonistic genetic interaction between Su(fu) and Hh-activated Fu. Thus, we conclude that S2R+ cells can produce both reliable Hh-dependent posttranslational modifications and transcription responses if core pathway components are sufficiently expressed together with Ci.

**References**


1. Lum L, Zhang C, Oh S, Mann RK, Kessler von DP, et al. (2003) Hedgehog signal transduction via Smoothened association with a cytoplasmic complex scaffolded by the atypical kinesin, Costal-2. Mol Cell 12: 1261–1274.

2. Denef N, Neubüser D, Perez L, Cohen SM (2000) Hedgehog induces opposite changes in turnover and subcellular localization of patched and smoothened. Cell 102: 521–531.

3. Thérond PP, Knight JD, Kornberg TB, Bishop JM (1996) Phosphorylation of the fused protein kinase in response to signaling from hedgehog. Proc Natl Acad Sci USA 93: 4224–4228.
